# miR825-5p targets the TIR-NBS-LRR gene *MIST1* and down-regulates basal immunity against *Pseudomonas syringae* in Arabidopsis

**DOI:** 10.1093/jxb/erab354

**Published:** 2021-07-30

**Authors:** Diego López-Márquez, Ángel Del-Espino, Nieves López-Pagán, Edgar A Rodríguez-Negrete, Ignacio Rubio-Somoza, Javier Ruiz-Albert, Eduardo R Bejarano, Carmen R Beuzón

**Affiliations:** 1 Instituto de Hortofruticultura Subtropical y Mediterránea ‘La Mayora’, Universidad de Málaga-Consejo Superior de Investigaciones Científicas (IHSM-UMA-CSIC), Depto. Biología Celular, Genética y Fisiología , Málaga, Spain; 2 Molecular Reprogramming and Evolution (MoRE) Lab, Centre for Research in Agricultural Genomics (CRAG), Carrer Vall Moronta Edifici CRAG, 08193, Barcelona, Spain; 3 Cardiff University, UK

**Keywords:** Arabidopsis, gene silencing, miRNA, phasiRNAs, plant immunity, *Pseudomonas syringae*, PTI, resistance genes

## Abstract

Plants encode numerous intracellular receptors known as nucleotide-binding leucine-rich repeat receptors (NLRs) that recognize pathogen-derived effectors or their activity to activate defenses. miRNAs regulate NLR genes in many species, often triggering the production of phased siRNAs (phasiRNAs). Most such examples involve genes encoding NLRs carrying coiled-coil domains, although a few include genes encoding NLRs carrying a Toll/interleukin-1 domain (TNL). Here, we characterize the role of miR825-5p in Arabidopsis, using a combination of bioinformatics, transgenic plants with altered miRNA levels and/or reporters, small RNAs, and virulence assays. We demonstrate that miR825-5p down-regulates the TNL *MIST1* by targeting for endonucleolytic cleavage the sequence coding for TIR2, a highly conserved amino acid motif, linked to a catalytic residue essential for immune function. miR825-5p acts as a negative regulator of basal resistance against *Pseudomonas syringae*. miR825-5p triggers the production from *MIST1* of a large number of phasiRNAs that can mediate cleavage of both *MIST1* and additional TNL gene transcripts, potentially acting as a regulatory hub. miR825-5p is expressed in unchallenged leaves and transcriptionally down-regulated in response to pathogen-associated molecular patterns (PAMPs). Our results show that miR825-5p, which is required for full expression of PAMP-triggered immunity, establishes a link between PAMP perception and expression of uncharacterized TNL genes.

## Teaser

miR825-5p down-regulates immunity against *Pseudomonas syringae* and mediates cleavage of the sequence encoding the conserved motif within the *MIST1* Toll/interleukin-1 (TIR) domain, triggering production of phased siRNAs that cleave additional TIR-carrying genes.

## Introduction

Plants possess complex immune systems that effectively protect them from the majority of pathogens within the environment. The functioning of these systems relies on a battery of cell surface and intracellular receptors that alert plants of incoming threats, leading to the activation of defense responses capable of hindering disease development (Cui *et al.*, 2015; Couto and Zipfel, 2016; Zhang *et al.*, 2017). Cell surface receptors [pattern recognition receptors (PRRs)] mediate perception of conserved molecules known as pathogen-associated molecular patterns (PAMPs), signaling the activation of basal resistance, known as PAMP-triggered immunity (PTI) (Monaghan and Zipfel, 2012). Intracellular immune receptors detect virulence factors (effectors) delivered by pathogens into the host cell, triggering a rapid and intense defense known as effector-triggered immunity (ETI) (Cui *et al.*, 2015). Most intracellular receptors belong to a large family of proteins known as NOD-like receptor (NLR; also known as nucleotide-binding leucine-rich repeat receptor) proteins. The NLR protein structure usually includes: a variable N-terminal domain, a nucleotide-binding domain (NB-ARC), and a leucine-rich repeat domain (LRR) (Meyers *et al.*, 2003; Cui *et al.*, 2015; Zhang *et al.*, 2017). NLRs include two major subfamilies: those displaying a Toll/interleukin-1 (TIR) N-terminal domain (TNLs), and those with an N-terminal domain resembling a coiled-coil (CC) domain (CNLs) (Jubic *et al.*, 2019). TNLs and CNLs typically engage the ETI machinery through different regulators of plant defense (Feys *et al.*, 2001; Wiermer *et al.*, 2005; Rietz *et al.*, 2011; Wagner *et al.*, 2013; Cui *et al.*, 2017).

Activation of NLR-mediated pathways is tightly controlled in the absence of the pathogen since constitutive activation often causes deleterious effects (Tian *et al.*, 2003; Korves and Bergelson, 2004; Ariga *et al.*, 2017; Karasov *et al.*, 2017). NLR protein production can be regulated at the transcriptional, post-transcriptional, translational, and post-translational levels. Twenty-one and twenty-two nucleotide long (21 and 22 nt) small RNAs (sRNAs) control NLR production at the post-transcriptional level. These sRNAs bind to their target mRNA by base pairing, reducing protein expression by either altering mRNA stability or inhibiting translation. This process, known as post-transcriptional gene silencing, engages proteins from the Argonaute (AGO) family (Axtell, 2013; Bologna and Voinnet, 2014). Twenty-two nucleotide long miRNAs play a central role as suppressors of NLR-encoding mRNAs (Zhai *et al.*, 2011). This type of miRNA can trigger the conversion of their mRNA targets into dsRNA by RNA-dependent RNA polymerase 6 (RDR6). These dsRNAs are processed by DICER-LIKE (DCL) 4 to generate 21 nt siRNAs, which are often phased with respect to the miRNA cleavage site, known as phasiRNAs (Chen *et al.*, 2010; Cuperus *et al.*, 2010; Fei *et al.*, 2013).

The first phasiRNAs which were characterized are *trans*-acting RNAs (tasiRNAs). tasiRNAs are generated by miRNA-directed cleavage of long non-coding TAS transcripts (Vazquez *et al.*, 2004; Allen *et al.*, 2005; Yoshikawa *et al.*, 2005; Felippes and Weigel, 2009), and act on secondary target genes, silencing their expression. Secondary siRNAs can also be generated from the transcripts of coding genes such as NLR genes (Li *et al.*, 2012; Shivaprasad *et al.*, 2012; Boccara *et al.*, 2014; Deng *et al.*, 2018; Cui *et al.*, 2020). The 22 nt miRNAs trigger production of siRNAs from NLR genes in several plant families (Li *et al.*, 2012; Shivaprasad *et al.*, 2012; Fei *et al.*, 2013; Boccara *et al.*, 2014; Deng *et al.*, 2018; Cui *et al.*, 2020). However, the potential *trans-*acting function of these siRNA has not often been experimentally confirmed. While this regulatory mechanism seems prevalent in plant families such as *Solanaceae* or legumes, it is absent in *Poaceae* and infrequent in *Brassicaceae* (Fei *et al.*, 2013). phasiRNAs generated from >100 NLR loci in *Medicago*, and some of the non-abundant phasiRNAs generated by miR472 in Arabidopsis act in *cis*, targeting transcripts from the miRNA target genes from which they are generated (Zhai *et al.*, 2011; Boccara *et al.*, 2014). phasiRNAs from *Medicago* and *Solanaceae* have been demonstrated to act in *trans*, generating degradome tags from the transcripts of secondary target genes (Zhai *et al.*, 2011; Deng *et al.*, 2018). *Trans*-silencing has been also demonstrated for *BraCP24* in *Brassica napus*, which is targeted by a phasiRNA generated from *BraTIR* upon miR1885-directed cleavage (Cui *et al.*, 2020). *BraTIR* does not encode a TNL but rather a sequence-related TIR-containing protein. *BraCP24* transcript levels display negative correlation with levels of miR1885, a rare example of such a correlation between miRNA and a secondary siRNA target.

NLR-regulating miRNAs typically target sequences encoding conserved protein motifs, with the P-loop-coding sequence being the most prevalent (Fei *et al.*, 2013; Zhang *et al.*, 2016). *Solanaceae* miR482/2118 and Arabidopsis sequence-related miR472 target the sequence coding for the P-loop, present in both TNLs and CNLs, although they display a strong preference for CNL genes (Shivaprasad *et al.*, 2012; Boccara *et al.*, 2014; Su *et al.*, 2018; Canto-Pastor *et al.*, 2019). In the absence of defense activation, altered levels of miR472 or miR482 lead to slight to mild changes of transcript levels of target genes (Shivaprasad *et al.*, 2012; Boccara *et al.*, 2014; Canto-Pastor *et al.*, 2019). Upon defense activation, transgenic plants with altered miR482/2118a or miR472 levels display bigger differences in target mRNA accumulation. For miR482/2118, the absence of a correlation between targeting scores and mRNA changes led the authors to propose that translational inhibition is involved (Shivaprasad *et al.*, 2012; Boccara *et al.*, 2014; Canto-Pastor *et al.*, 2019). miR482 and miR472 trigger the production of siRNAs with a low degree of phasing (Shivaprasad *et al.*, 2012; Boccara *et al.*, 2014; Su *et al.*, 2018; Canto-Pastor *et al.*, 2019). miR472 silencing of target mRNAs is amplified by *cis*-silencing by secondary siRNAs (Boccara *et al.*, 2014). miR2118b primarily targets the NLR-related *TAS5* gene, triggering the production of siRNAs that may potentially target CNL and TNL genes (Shivaprasad *et al.*, 2012; Boccara *et al.*, 2014; Canto-Pastor *et al.*, 2019). However, no correlation has been reported between changes in miR2118b levels and changes in transcript levels for secondary target genes, also leading to the proposal that miR2118b–*TAS5*–siRNA-mediated silencing of secondary targets might take place through translational inhibition. miR472 and miR482 down-regulate basal immunity against the bacterial pathogen *Pseudomonas syringae* in Arabidopsis and tomato, respectively, and this down-regulation is alleviated upon infection (Shivaprasad *et al.*, 2012; Boccara *et al.*, 2014). miR472-dependent down-regulation is also released upon treatment with flg22, an immune elicitor peptide from bacterial flagellin and a major activator of PTI (Boccara *et al.*, 2014; Su *et al.*, 2018). miR482-dependent down-regulation can be lifted by type III secretion system (T3SS)-mediated translocation of bacterial effectors into the host cell (Shivaprasad *et al.*, 2012).

In Arabidopsis, in addition to miR472 regulation of CNL genes, levels of 21 nt and 22 nt miRNAs generated from opposite arms of the *MIR825* transcript display negative correlations with mRNA levels of several Arabidopsis genes, including TNL genes (Niu *et al.*, 2016), and with resistance to *P. syringae* and *Botrytis cinerea* (Niu *et al.*, 2016; Nie *et al.*, 2019). Here, we focus on the molecular characterization of miRNA825-5p, the 22 nt miRNA produced from *MIR825*. miR825-5p, originally identified as passenger miRNA (miR825*), has been proposed as a potential trigger of phasiRNAs (Chen *et al.*, 2010). Here, we experimentally demonstrate that miR825-5p targets the Arabidopsis *AT5G38850* TNL gene (named here *MIST1* for *microRNA-silenced TNL1*), silencing its expression through endonucleolytic cleavage of its transcripts. miR825-5p targets a sequence coding for a highly conserved amino acid motif, TIR2, located within the TIR domain and adjacent to the catalytic residue for recently demonstrated NAD^+^-cleaving enzymatic activity, essential for TNL function (Wan *et al.*, 2019). miR825-5p triggers the production of a large amount of phasiRNAs from *MIST1* transcripts, the highest amount for phasiRNAs of an NLR gene in Arabidopsis, in adult leaves under basal conditions. Degradome tags reveal *cis*-targeting of *MIST1* transcript by *MIST1*-derived phasiRNAs, as well as *trans*-targeting of additional uncharacterized TNL-encoding secondary target genes. In addition, we demonstrate that miR825-5p represses basal disease resistance against *P. syringae.* Finally, we also show that miRNA825-5p is down-regulated at the transcriptional level in response to PAMPs, in a salicylic acid (SA)-dependent and partially T3SS-independent manner. Our results characterize a regulatory network where miR825-5p functions as a negative regulator of basal immunity, with the target gene *MIST1* acting as a phasiTNL-producing hub, which potentially includes more TNL genes in Arabidopsis. Our results detail a mechanism that reinforces the link between PAMP perception and TNL activation.

## Materials and methods

### Plant material

*Arabidopsis thaliana* plants were grown in soil, or Murashige and Skoog (MS) plates without sucrose, at 21 °C with a photoperiod of 8 h light/16 h darkness with a light intensity of 200 µmol m^–2^ s^–1^. Seeds were stratified for 2 d at 4 °C. For MS growth, seeds were surface-sterilized with a mixture of ethanol and bleach for 15 min and washed three times with ethanol. *Nicotiana benthamiana* plants were grown in soil at 22 °C with a photoperiod of 16 h light/8 h darkness and the same light intensity.

### Cloning procedures

DNA fragments were amplified by PCR using Q5 High-Fidelity DNA polymerase (NEB, USA), and cloned into pGEM-T (Promega, USA) or pENTR (Thermo Fisher Scientific, USA). pGEM-T clones were subcloned into destination vectors by restriction–ligation cloning, while pENTR clones were subcloned using Gateway LR Clonase II Enzyme mix (Invitrogen, USA). Gotaq Flexi DNA Polymerase (Promega, USA) was used for PCR-based cloning confirmation or plant genotyping. Primers are listed in Supplementary Table S1.

We used the WMD3 tool to design a 21 nt artificial miRNA against the common precursor for miR825-5p and miR825-3p (pri-miR825), complementary to the sequence of miR825-5p. This 21 nt sequence of interest was introduced into the miR319 precursor, substituting miR319, as recommended by WMD3. The resulting modified precursor was purified and ligated into *Eco*RV-digested pBlueScript II SK and confirmed by sequencing. The resulting plasmid was digested with *Sal*I and *Bam*HI (Takara, Japan), and the fragment containing the precursor was gel purified and cloned into the *Sal*I–*Bam*HI sites of pBINX′. The resulting plasmid, expressing the modified precursor under the control of a 2×35S promoter (anti825), was confirmed by sequencing. The same procedure was used to generate a vector overexpressing amiR825-5p. We designed the corresponding primers using the WMD3 tool, and then removed a nucleotide from the miRNA* region, in order to generate a 22/21 nt miRNA/miRNA* asymmetric pair. The resulting plasmid, expressing the modified precursor under the control of a 2×35S promoter, was also confirmed by sequencing. For overexpression of miR319, a fragment corresponding to the miR319 precursor sequence was directly obtained through *Sal*I and *Bam*HI (Takara, Japan) digestion of pRS300, followed by gel purification. The resulting DNA fragment was ligated into *Sal*I–*Bam*HI-digested pBINX′. The resulting plasmid, expressing the modified precursor under the control of a 2×35S promoter, was confirmed by sequencing. To generate 2×35s::STTM825-5 p, a pair of long, partially complementary primers, containing *Hpa*I (primer F) and *Bam*HI (primer R) sites, was designed. These primers were used to generate STTM825-5p, which contains two copies of miRNA825-5p target site, each one with a 3 nt bulge inserted, with both copies separated by a 48 nt spacer. The *Hpa*I–*Bam*HI-digested PCR fragment was gel purified and ligated into *Hpa*I–*Bam*HI-digested pBINX′, placing the construct under the control of a 2×35S promoter, and checked by sequencing. To generate the construct containing the miR825 promoter, 2 kb of the miRNA promoter region was PCR-amplified using primers containing *Not*I (primer F) and *Asc*I (primer R) sites. The gel-purified and *Not*I (Takara, Japan)–*Asc*I (Thermo Scientific, USA)-digested fragment was column-purified and ligated into the same sites of pENTR (Invitrogen, USA), and checked by sequencing. Finally, the promoter region was introduced into plasmid pMDC111 by an LR reaction (Invitrogen, USA). The 5′-untranslated region (UTR) and the entire ORF for AT5G38850 (from the ATG to the last codon before the stop codon) was amplified. The stop codon was not included to allow for the generation of C-terminal tag fusions. Primers included a *Not*I (forward primer) or an *Asc*I (reverse primer) site. The gel-purified DNA fragment generated by PCR was digested with *Not*I and *Asc*I, column-purified, ligated into the same sites of pENTR (Invitrogen, USA), and checked by sequencing. Finally, the AT5G38850 ORF was introduced by an LR reaction (Invitrogen, USA) into plasmid pEG103, to generate a C-terminal fusion to green fluorescent protein (GFP). A mutant version of the AT5G38850 ORF lacking the miRNA cleavage site (m-AT5G38850) was generated using a pair of primers designed to alter the miR825-5p target site by inverted PCR, using pENTR-AT5G38850-ORF as a template, and the NZYMutagenesis Kit (NZYTech, Portugal) following the manufacturer’s instructions. After sequence confirmation, the m-AT5G38850 ORF was introduced by an LR reaction (Invitrogen, USA) into plasmid pEG103, to generate a C-terminal fusion to GFP. For generating a *trans*-silencing reporter (MIGS825-5pTS), the fragment of the *A. thaliana* AGAMOUS gene (AG) used by Felippes *et al.* (2012) was amplified by PCR using a forward primer encompassing the miR825-5p target site present in *MIST1* instead of the miR173 target site. Primers included a *Not*I (forward primer) or an *Asc*I (reverse primer) site to allow *Not*I–*Asc*I cloning into pENTR (as described above). Once confirmed by sequencing, the fragment was introduced by an LR reaction (Invitrogen, USA) into plasmid pGWB2, placing it under the control of the plasmid-borne 35S promoter.

All the cloning procedures were performed using the Q5 High-Fidelity DNA polymerase (NEB, USA) for PCRs, the Wizard SV Gel and the PCR Clean-Up System (Promega, USA) for DNA purifications (either from agarose gels or after enzymatic reactions), and T4 DNA ligase (Takara, Japan) for ligations. All reactions and procedures were carried out following the manufacturers’ instructions.

### RNA isolation and RT–qPCRs

Total RNA was extracted from 100 mg of plant tissue (ground in liquid nitrogen) using TRISURE (Bioline, UK). cDNA was generated from 1 µg of total RNA, pre-treated with DNase I (Takara, Japan). For semi-quantitative PCR, we used 1 µl of cDNA, and 20–28 cycles to avoid saturation.

A 2 µl aliquot of a 1/5 cDNA dilution was used for reverse transcription–quantitative PCRs (RT–qPCRs) in a reaction containing 5 µl of SsoFast EvaGreen (Bio-Rad, USA), 0.5 µl of each 10 µM forward and reverse primer, and 2 µl of H_2_O, on CFX96 or CFX384 thermocyclers (Bio-Rad), with a 1 min denaturing step at 95 °C, and 45 cycles of 10 s at 95 °C and 15 s at 60 °C. ACT2 was used as internal control. Supplementary Tables S2 and S3 show ACT2 expression normalized to UBQ5 as a second normalizer control gene. Relative expression was calculated using the 2^−ΔΔCt^ method (Livak and Schmittgen, 2001). We used stem–loop RT–qPCR to quantify mature miRNAs (Varkonyi-Gasic, 2016). A pulsed reverse transcription reaction (16 °C for 30 min, followed by 60 cycles at 30 °C 30 s, 42 °C 30 s, and 50 °C 1 min; and 5 min at 85 °C) (Varkonyi-Gasic, 2016) was performed using the Revert Aid First Strand cDNA Synthesis Kit (Thermo Fisher Scientific, USA) with specific primers and oligo(dT), and CFX96 or CFX384 (Bio-Rad) thermocyclers.

### Small RNA northern blots

Total RNA was used for hybridizations as described (Tomassi *et al.*, 2017). For probe labeling, a DNA oligonucleotide reverse complement to U6 or miRNA was 3′ end-labeled with digoxigenin-11-ddUTP (Sigma, USA) using a terminal deoxynucleotidyl transferase (TdT; ThermoFisher Scientific, USA) in a reaction containing 20 U of TdT, 10 μl of 5× TdT reaction buffer, 5 μl of DNA primer (1 μM), 2.5 μl of digoxigenin-11-ddUTP (10 μM), and H_2_O to 50 μl. The reaction was incubated for 40 min at 37 °C and added to the hybridization solution without further purification. For miR825-5p detection, a dual-labeled (5′ and 3′ DIG) LNA (locked nucleic acid) probe was used (Qiagen, Germany). For secondary siRNA detection, a fragment of *MIST1* was PCR-amplified using Q5 DNA polymerase (NEB, USA) with MIST1_PhasiRNA_ProbeF and MIST1_PhasiRNA_ProbeR. A 25 ng aliquot of the gel-purified PCR product was used in a random priming reaction containing: 4 U of Klenow fragment (Takara, Japan), 5 μl of 10× Klenow reaction buffer, 12 μl of random hexamers (100 μM), 5 μl of 10× DIG DNA labeling mx (Sigma, USA), and H_2_O to 50 μl.

### Protein extraction and western blot

A 100 µg aliquot of leaf tissue, frozen in liquid nitrogen, was mechanically disrupted into 100 µl of Laemmli buffer (Laemmli, 1970), then centrifuged twice at 20 000 *g* for 10 min at 4 °C to remove debris and extract proteins. Aliquots of 10 µg per sample were typically resolved on 10–12% acrylamide SDS–PAGE gels and transferred onto PVDF Immobilon-P membranes (Millipore, USA). Western blots were performed using standard methods (Sambrook and Russell, 2001) with the antibodies listed in Supplementary Table S4. Protein concentration was determined by Bradford protein assay, and membranes were developed using Clarity Western ECL Substrate (Bio-Rad, USA).

### Bacterial assays

Bacterial *in planta* assays were carried out using the strains listed in Supplementary Table S5. Colonies from LB (Bertani, 1951) plates were incubated for 2 d at 28 °C. For RT–PCR or semi-quantitative PCR analysis, a 10 mM MgCl_2_ bacterial suspension at 5×10^7^ colony-forming units per ml (cfu ml^–1^) was used to pressure-infiltrate Arabidopsis adult leaves. Inoculations for bacterial proliferation assays were carried out by infiltrating three leaves per plant with 5×10^4^ cfu ml^–1^ suspensions. Four days post-inoculation (dpi), we collected one 10 mm diameter leaf disk from each inoculated leaf per plant, homogenizing the three disks together into 10 mM MgCl_2_ to generate a biological replicate. Serial dilutions were plated onto LB with 2 µg ml^–1^ cycloheximide, and incubated at 28 °C for cfu cm^–2^ determination.

Transient expression assays in *N. benthamiana* were performed as described (Rufián *et al.*, 2015) by infiltrating 5-week-old plants with *Agrobacterium* C58C1 carrying the corresponding binary plasmids (Supplementary Table S6). Samples were taken at 2–3 dpi.

### Generating Arabidopsis transgenic lines

*Arabidopsis thaliana* transgenic lines (Supplementary Table S7) were generated by transformation with *Agrobacterium* cultures carrying the corresponding binary plasmids (Supplementary Table S6), following the standard floral dipping procedure (Clough and Bent, 1998). Transformants were selected into MS plates supplemented with kanamycin (50 μg ml^–1^) or hygromycin (40 μg ml^–1^), and the presence of the transgene was confirmed by PCR.

### Basal defense response elicitation

A 100 nM solution of flg22 immunogenic flagellin peptide (GenScript, USA), or a 0.2% solution of chitin (method2 from Souza *et al.*, 2009), was pressure-infiltrated into adult plant leaves. Each assay included plants infiltrated with water as mock treatment. Samples were frozen in liquid nitrogen and stored at –80 °C.

### Mitogen-activated protein kinase activation assays

For mitogen-activated protein kinase (MPK) activation assays, 12-day-old *A. thaliana* seedlings grown in MS plates (four per sample) were transferred into liquid MS and incubated for 24 h, and then transferred to 12-well plates containing a 100 nM solution of flg22. Samples collected were ground in liquid nitrogen, and proteins were extracted in a buffer containing 100 mM Tris–HCl, 150 mM NaCl, and 1× Halt Phosphatase Inhibitor Cocktail (Thermo Fisher Scientific, USA). Proteins were subjected to western hybridization as described above, using anti-MPK or anti-tubulin antibodies (Supplementary Table S4).

### Quantification of reactive oxygen species (ROS)

Arabidopsis plants were grown in soil for 2–3 weeks. Two leaf disks were taken per plant with a cork borer (3.8 mm diameter), transferred into a 96-well plate containing 100 µl of water per well, and incubated for 24 h at room temperature. Water was replaced by 100 µl of a solution containing 17 µg ml^–1^ Luminol (Sigma-A8511, USA), 10 µg ml^–1^ horseradish peroxidase (HRP) (Sigma-P6782, USA), and 100 nM flg22. Light emission was measured in a GloMax 96 Microplate Luminometer (Promega, USA). At least 16 leaf disks were taken by treatment (*n*≥16).

### Bioinformatic analysis

Databases, libraries, software, and bioinformatic methods used in this work are detailed in Supplementary Tables S8 and S9.

## Results

### miR825-5p is a negative regulator of immunity against *P. syringae*

**Fig. 1.**
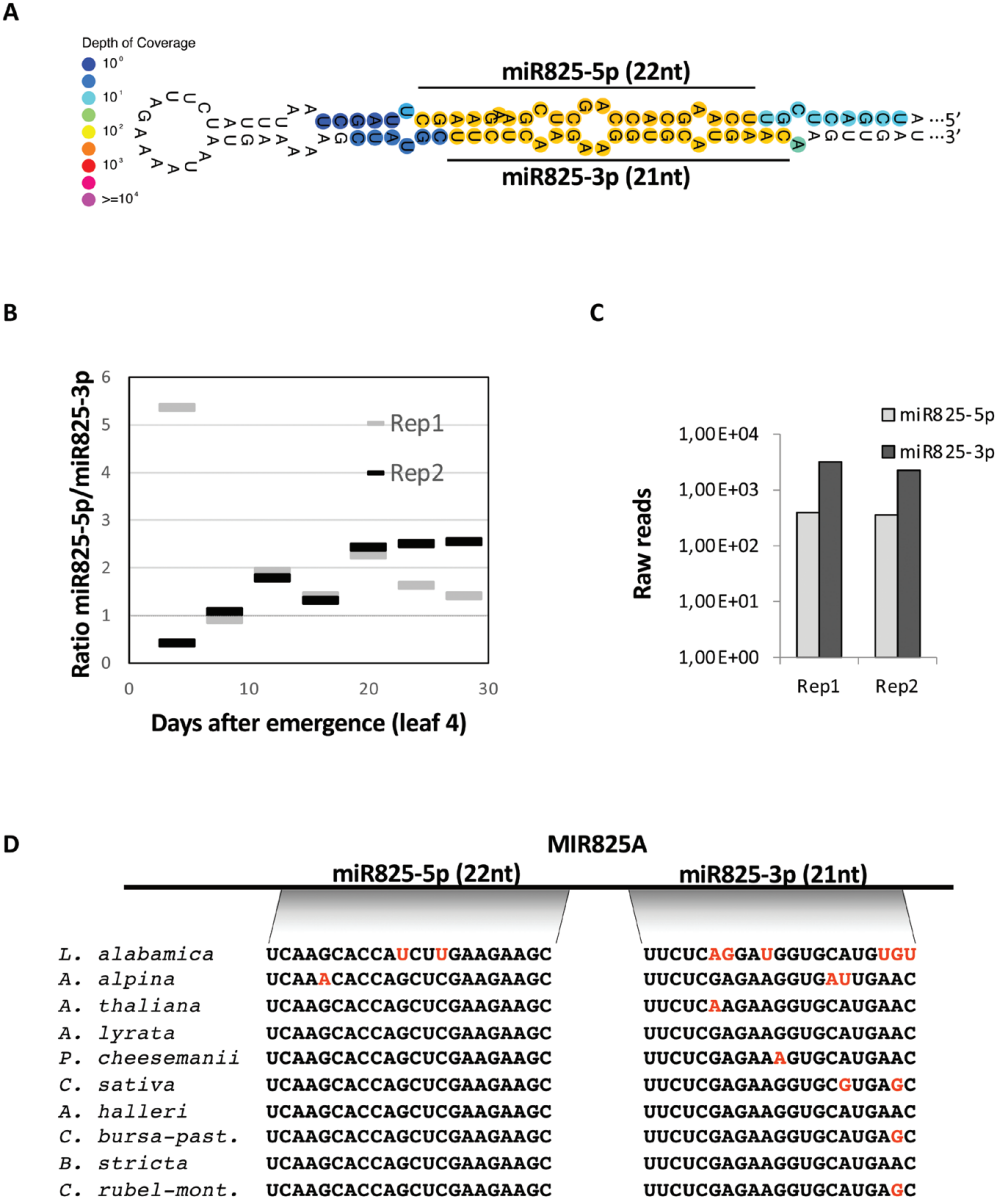
miR825-5p is a candidate regulator of TNLs. (A) Two different miRNAs, one of 21 nt and the other of 22 nt (here named miR825-3p and miR825-5p) are generated with good coverage from opposite arms of the pri-miRNA duplex expressed from *MIR825* (SRR2079800). (B) Ratio between levels of miR825-5p and miR825-3p in leaf four on different days after emergence in Col-0 plants, as stored in public databases (PRJNA186843). Two replicate experiments are shown. (C) The graph shows raw reads of miR825-5p and miR825-3p pulled down as part of AGO1 complexes. The data were obtained from GSM2787769 and GSM2787770 public databases. (D) Sequence comparisons between 21 nt miR825-3p and 22 nt miR825-5p encoded in different Brassica species show that miRNA825-5p is conserved to a higher degree than miRNA825-3p.

While analyzing the overlap between transcriptomic responses to *P. syringae* and gene silencing in Arabidopsis, serendipity led us to look into the *MIR825* locus. Two miRNAs, one of 21 nt (miR825) (Rajagopalan *et al.*, 2006) and the other of 22 nt (miR825*) (Chen *et al.*, 2010) are generated with good coverage from opposite arms of the pri-miRNA expressed from *MIR825* (Fig. 1A). Target prediction rendered only three putative targets for 21 nt miR825 (miR825-3p, see below), annotated as poly(A)-binding protein- and ubiquitin carboxyl-terminal hydrolase-encoding genes (Supplementary Fig. S1A; Supplementary Table S10). Numerous targets were predicted for 22 nt miR825* (miR825-5p, see below), displaying lower hybridization energies and better pairing, which were mostly annotated as uncharacterized TNL genes (Supplementary Fig. S1A; Supplementary Table S10). Alternative software (Dai and Zhao, 2011) gave similar results (Supplementary Table S10). Predicted binding sites for 22 nt miR825 TNL targets map within the sequence coding for the TIR domain (Supplementary Fig. S1B) in particular the highly conserved TIR2 motif (Meyers *et al.*, 1999). TIR2 immediately adjoins a catalytic glutamic acid residue for the recently demonstrated NAD^+^-cleaving enzymatic activity, which is essential for TNL function (Wan *et al.*, 2019).

Primary miRNAs typically display higher conservation and accumulation than passenger miRNAs, often destined for degradation (Voinnet, 2009; Achkar *et al.*, 2016; Yu *et al.*, 2017). We analyzed levels of *MIR825*-derived miRNAs using an available dataset (Bioproject PRJNA186843). The 22 nt miR825-5p consistently accumulates to similar or higher levels than 21 nt miR825-3p (Fig. 1A, B). Searches within AGO1-bound sRNA libraries (Supplementary Table S9) showed that both miRNAs are consistently pulled down in association with AGO1 complexes with good coverage, supporting their participation in gene silencing (Fig. 1C). *MIRNA825* has been classified as a Class V miRNA (Eamens *et al.*, 2009). Class V miRNA duplexes are bilaterally symmetrical with regards to thermostability at the terminus of both duplex strands, resulting in equal accumulation of both miRNAs. Our results support the *MIR825* duplex as a Class V miRNA, with processing of the *MIRNA825* duplex leading to two functional miRNAs, making the designation of miR825/miR825* outdated. In keeping with the designation used in other Brassica species (e.g. broccoli, Tian *et al.*, 2014; *Arabidopsis lyrata*, miRBase Release 21), we have updated their names in Arabidopsis to miR825-5p (22 nt) and miR825-3p (21 nt). *MIRNA825* is mainly restricted to Brassica species, with miR825-5p displaying very high conservation (Fig. 1D) (Zhang *et al.*, 2016).

**Fig. 2.**
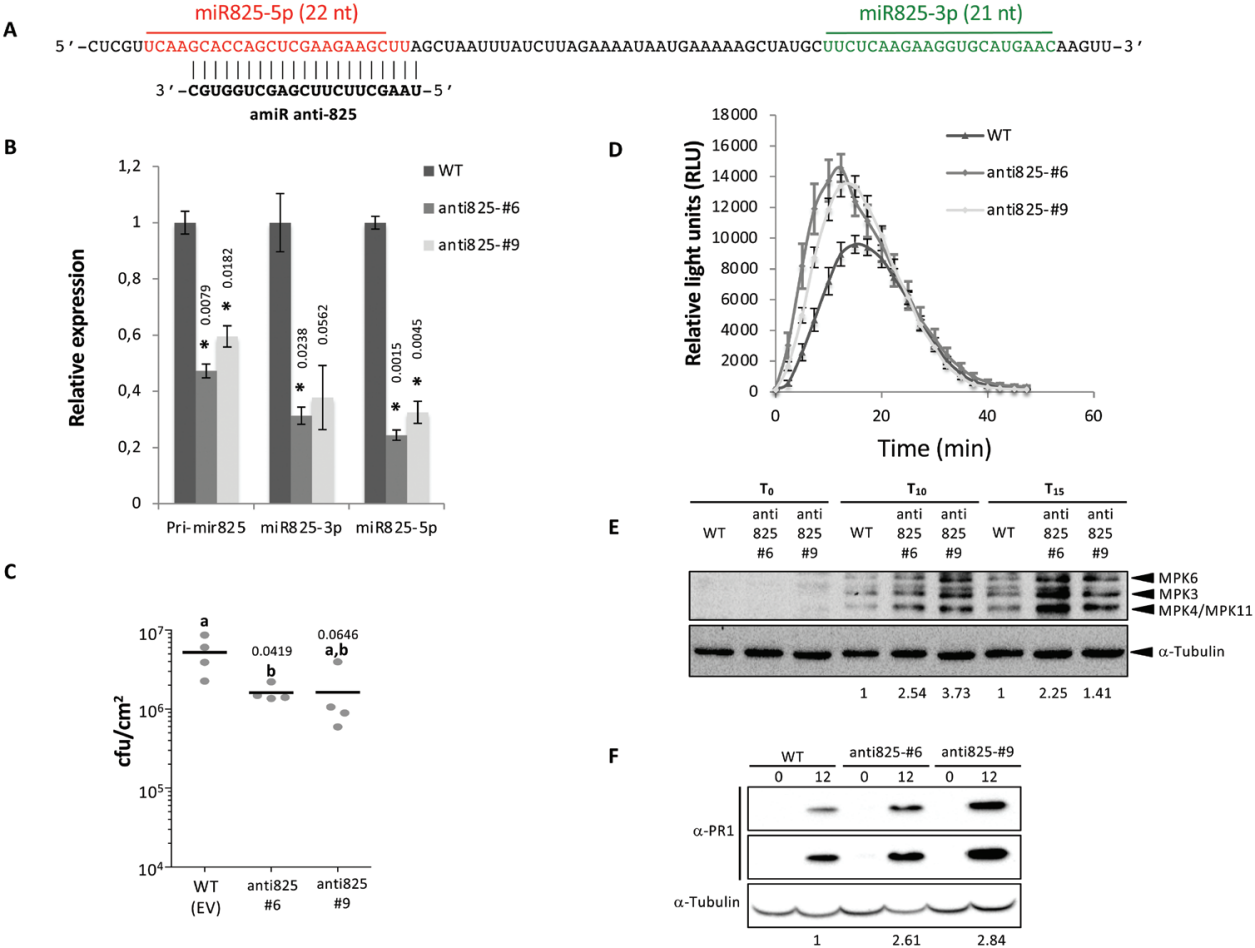
pri-miR825 has a negative impact on PTI. (A) Sequence of pri-miR825 indicating the target for amiR anti825. (B) Plants expressing amiR anti825 display significantly reduced precursor levels, as well as significantly reduced levels of the mature forms of miR825-5p and miR825-3p compared with wild-type Col-0 plants (WT). Asterisks indicate that the results are significantly different from WT plants, as established by a Student’s *t*-test (*P*<0.05). Numbers above the bars indicate *P*-values. Error bars correspond to the SE. Similar results were obtained in two biological replicates. (C) Bacterial multiplication assay in WT or anti825 lines: leaves were inoculated by infiltration with a solution of 5×10^4^ cfu ml^–1^ of *P. syringae* DC3000. Samples were taken 4 days post-inoculation and plated. Bacterial counts are shown. Mean values are shown for each plant genotype, although individual values are also represented. Mean values marked with the same letter were not significantly different from each other as established by Student’s *t*-test (*P*<0.05). Similar results were obtained in two biological replicates. (D) ROS production at different time points after treatment with 100 nM flg22 of WT or anti825 lines. Similar results were obtained in two biological replicates. (E) Western blot analysis showing levels of phosphorylated mitogen-activated protein kinases (MPK3, MPK4, MPK6, and MPK11) after treatment with 100 nM flg22 of wild-type (WT) or anti825 lines at three different time points (0, 10, or 15 min post-flg22 treatment). Anti-tubulin was used for normalization. Numbers below the blot indicate fold differences between MPK/tubulin signal ratios calculated using ImageJ (http://imagej.nih.gov/ij/) in anti825 lines and the ratio obtained for WT plants at each time point. Similar results were obtained in two biological replicates. (F) Western blot analysis showing PR1 protein levels in WT or anti825 plants after inoculation with 5×10^7^ cfu ml^–1^ of *P. syringae* DC3000 expressing effector AvrRpt2 from a plasmid under a constitutive *nptII* promoter. Samples were taken at 0 or 12 h post-inoculation. Anti-tubulin was used as loading control. Similar results were obtained in two biological replicates.

Arabidopsis plants simultaneously overexpressing or silencing both miR825-5p and miR825-3p have been reported to affect *P. syringae* colonization (Niu *et al.*, 2016). To characterize the defense processes affected, we engineered plants to express an artificial miRNA (amiR anti825; (Supplementary Fig. S2A) (Schwab, 2006; Ossowski *et al.*, 2008), where the precursor for miR319 is modified to target and silence pri-miR825 (Fig. 2A) (Eamens *et al.*, 2011), and assayed them using *P. syringae* strains or an elicitor (Fig. 2). These plants display reduced levels of pri-miR825 and reduced levels of mature miR825-5p and miR825-3p, compared with the wild type, and no morphological or developmental differences (Fig. 2B). As expected, *MIR825*-silenced plants were more resistant to *P. syringae* DC3000 colonization (Fig. 2C). When these plants were treated with the immunogenic flagellar peptide flg22, they displayed enhanced responses in early markers of PTI activation such as higher ROS production (Fig. 2D), and activation of MPK (Fig. 2E). These plants also displayed increased accumulation of pathogenesis-related 1 protein (PR1) in response to inoculation with *P. syringae* DC3000 expressing the heterologous avirulence gene *avrRpt2* (Fig. 2F). Since none of the potential targets of *MIR825* miRNAs has been linked to AvrRpt2-mediated immunity, PR1 accumulation in *MIR825*-silenced plants may result from lifting of *MIR825* miRNA-dependent down-regulation of PTI. A recent report shows that simultaneous changes in the levels of miR825-3p and miR825-5p affect PTI markers during infection with *B. cinerea* in plants pre-treated with *Botrytis cereus* to trigger induced systemic resistance (ISR) (Nie *et al.*, 2019), but not without *B. cereus*-mediated ISR.

**Fig. 3.**
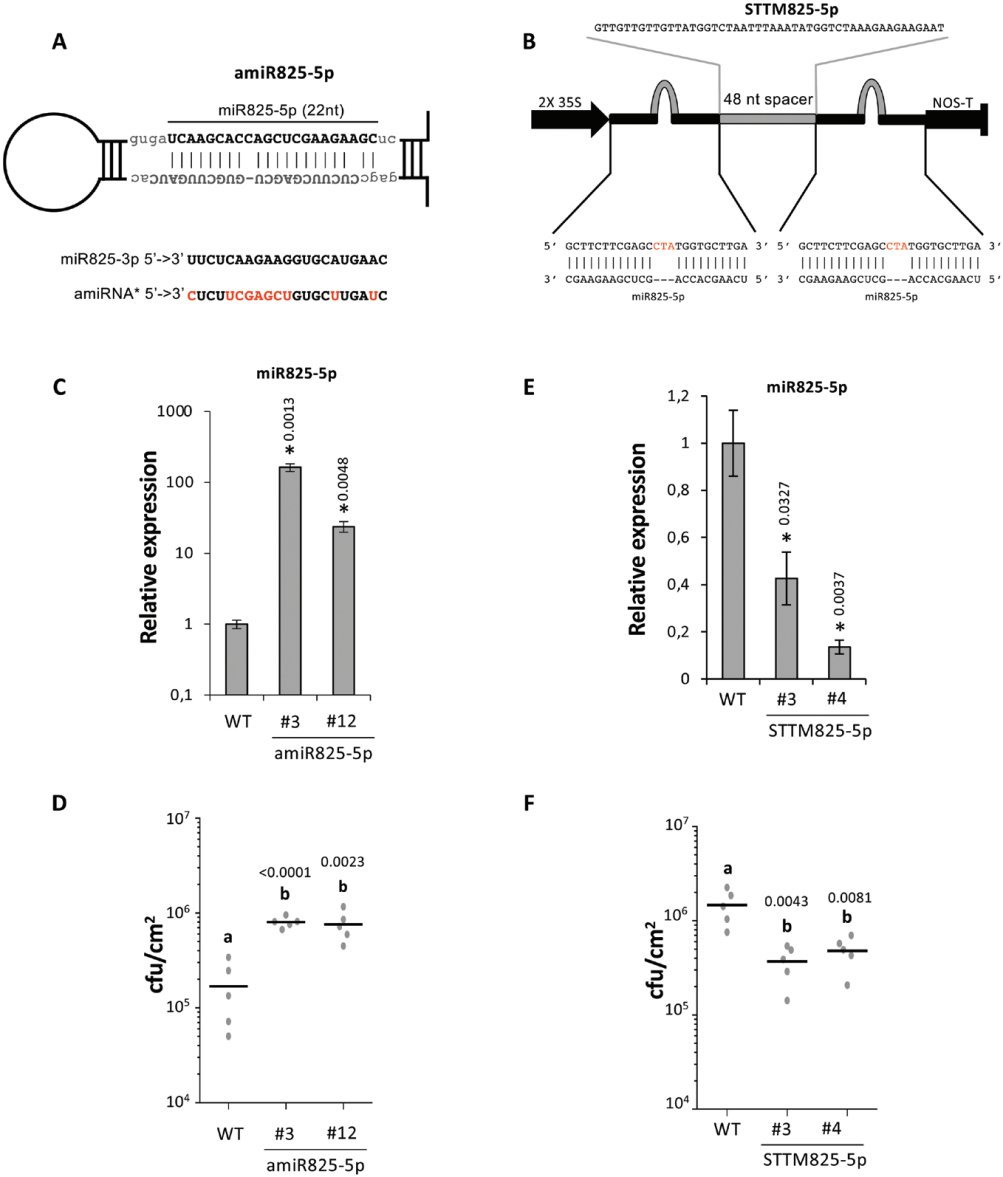
miR825-5p is a negative regulator of plant immunity against *P. syringae*. (A) Sequence and hairpin structure of amiR825-5p. A sequence comparison between miR825-3p and the passenger miRNA generated from this construct (amiRNA*) is shown below the hairpin. (B) Sequence and structure of the STTM825-5p construct. (C) Relative expression of miR825-5p in the wild type (WT) and two independent lines expressing amiR825-5p (#3 and #12). Asterisks indicate that the results are significantly different from WT plants, as established by a Student’s *t*-test (*P*<0.05). Error bars correspond to the SE. Numbers above bars indicate the *P*-value. Similar results were obtained in three biological replicates. (D) Bacterial colonization of the WT and two independent lines expressing amiR825-5p (#3 and #12). Similar results were obtained in three biological replicates. (E) Relative expression of miR825-5p in the WT and two independent lines expressing STTM825-5p (#3 and #4). Asterisks indicate that results are significantly different from WT plants, as established by a Student’s *t*-test (*P*<0.05). Error bars correspond to the SE. Numbers above bars indicate the *P*-value. Similar results were obtained in three biological replicates. (F) Bacterial colonization of the WT and two independent lines expressing STTM825-5p (#3 and #4). Similar results were obtained in three biological replicates. For bacterial colonization assays in (D) and (F), plants were inoculated by infiltration of a 5×10^4^ cfu ml^–1^ of *P. syringae* DC3000 solution. Samples were taken 4 d post-inoculation and plated. Bacterial counts are shown. Mean values are shown for each plant genotype, although individual values are also represented. Mean values marked with the same letter were not significantly different from each other as established by Student’s *t*-test (*P*<0.05). *P*-values are shown above the letters.

The nature of TNLs as pathogen receptors and their prevalence among miR825-5p predicted targets point to miR825-5p as directly involved in down-regulating plant immunity (Fig. 2). To investigate this, we generated *A. thaliana* transgenic plants with increased or reduced levels of mature miR825-5p using either artificial miRNAs or short tandem target mimic (STTM) technology, respectively (Fig. 3A, B) (Schwab, 2006; Yan *et al.*, 2012). These approaches achieve changes in the levels of a mature miRNA, without altering levels of an additional miRNA generated from the endogenous pri-miRNA duplex, in this case, miR825-3p (Schwab, 2006; Yan *et al.*, 2012). amiR825-5p transgenic lines displayed increased accumulation of miR825-5p (Fig. 3C) and decreased resistance to *P. syringae* (Fig. 3D). In turn, STTM825-5p lines, which accumulated reduced miR825-5p levels (Fig. 3E), displayed increased resistance (Fig. 3F). Transgenic lines with reduced levels of mature miR825-5p (Fig. 3E) recapitulated the levels of bacterial colonization (Fig. 3F) observed in lines with reduced levels of pri-miR825 (Fig. 2C). They also recapitulated the impact that changes in pri-miR825 have on activation of MPKs (Supplementary Fig. S2D). Although we cannot rule out that miR825-3p may redundantly contribute to plant defense against *P. syringae*, these results demonstrate that miR825-5p acts as a negative regulator of immunity against this pathogen.

### The *AT5G38850* TIR-NBS-LRR transcript is a target of miR825-5p

**Fig. 4.**
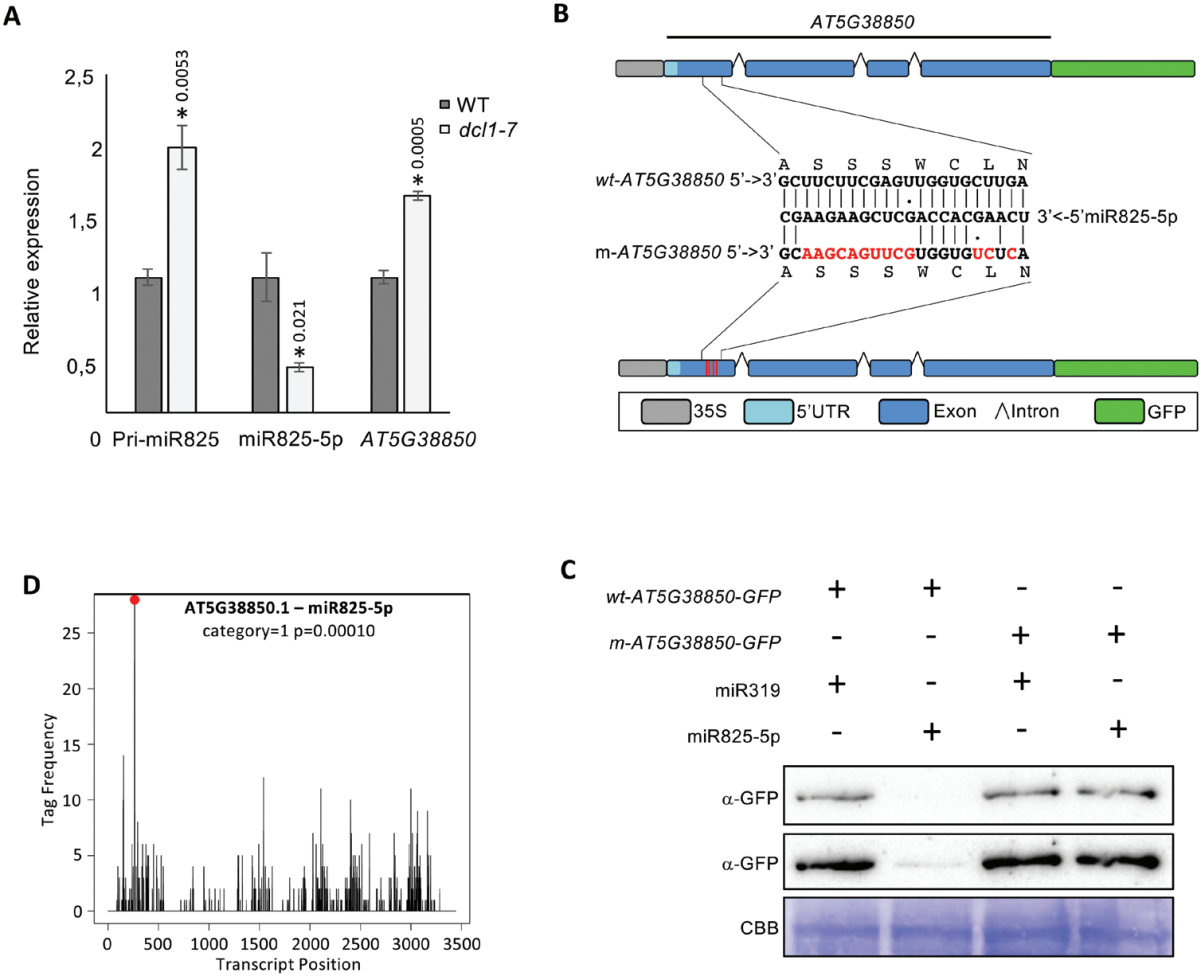
The TNL-encoding *AT5G38850* gene is a target for miR825-5p regulation. (A) The graph shows levels of pri-miR825, miR825-5p, and AT5G38850 mRNA in wild-type (WT) versus *dcl1-7* mutant plants. Asterisks indicate that results are significantly different from WT plants, as established by a Student’s *t*-test (*P*<0.05). Error bars correspond to the SE. Similar results were obtained in two biological replicates. (B) Gene fusion of AT5G38850 (includes its own 5′-UTR, exons, and introns) to the green fluorescent protein gene (*GFP*) ORF. The gene fusion is under the transcriptional control of a 35S constitutive promoter (wt-AT5G38850). The modified version carries mutations making the transcript generated no longer complementary to miR825-5p, without affecting protein coding (m-AT5G38850). (C) Western blot analysis using an anti-GFP antibody of *Nicotiana benthamiana* leaves transiently co-expressing either wt-AT5G38850 or m-AT5G38850, with either miR825-5p or unrelated miR319. Two exposure times are shown. Coomassie blue staining of the membrane is shown as a loading control. Similar results were obtained in three biological replicates. (D) T-plot showing density of the 5′ position of degradome tags corresponding to *MIST1* from library SRR10322040 (for graphs from the other libraries, see Supplementary Fig. S3). The red dot indicates degradome tags starting at the predicted target site for miR825-5p (slicing between the 10th and 11th nucleotides at the complementary target site relative to the miRNA). Category refers to PARE read abundance of that position (Addo-Quaye *et al.*, 2009). The *P*-value is indicated.

Since processing of the pri-miR825 depends on the activities of DCL1 and DCL3 (Fahlgren *et al.*, 2007; Vazquez *et al.*, 2008), we confirmed that accumulation of pri-miR825 increased, and levels of miR825-5p concomitantly decreased in a *dcl1-7* mutant (Fig. 4A). Conversely, transcript levels of *AT5G38850*, the top predicted target for miR825-5p (Supplementary Fig. S1; Supplementary Table S10), were significantly increased in *dcl1-7* plants. This supports previous reports of a negative correlation between simultaneously altered levels of miR825-3p/miR825-5p and *AT5G38850* levels (Niu *et al.*, 2016; Nie *et al.*, 2019). To obtain experimental evidence of miR825-5p targeting of *AT5G38850*, we generated a translational gene fusion of *AT5G38850* (5′-UTR, exons, and introns) to the *GFP* ORF, under the control of the *Cauliflower mosaic virus* (CaMV) 35S constitutive promoter (wt-*AT5G38850*; Fig. 4B). We also generated a modified version in which the miR825-5p target site has lost complementary to the miRNA, without altering the encoded amino acid sequence (m-*AT5G38850*; Fig. 4B). *Nicotiana benthamiana* leaves co-expressing wt-*AT5G38850-GFP* and miR825-5p accumulate very low levels of GFP, compared with those accumulated in leaves co-expressing the gene fusion and unrelated miR319 (Fig. 4C). GFP levels on control leaves expressing wt-*AT5G38850-GFP* and miR319 were similar to those detected in leaves co-expressing m-*AT5G38850-GFP* and either miR825-5p or miR319 (Fig. 4C). These results indicate that miR825-5p regulates protein accumulation by recognizing a complementary sequence within the *AT5G38850* transcript. Accordingly, levels of endogenous *AT5G38850* transcript negatively correlated with miR825-5p levels in transgenic plants displaying altered levels of this miRNA (Supplementary Fig. S2).

To seek evidence of miR825-5p-mediated endonucleolytic cleavage of *AT5G38850* mRNA in wild-type plants, we used CleaveLand4 to analyze available degradome (parallel analysis of RNA ends or PARE) libraries of Arabidopsis adult leaves (Supplementary Table S9) (Addo-Quaye *et al.*, 2008, 2009). We found a degradome tag accumulating significantly in three independent libraries, mapping to the miR825-5p predicted cleavage site on *AT5G38850* (Fig. 4D; Supplementary Fig. S3). As a control, we found degradome tags within the reported miR472 target gene *AT5G43740* (Supplementary Fig. S3). Degradome analysis provides experimental evidence indicating that miR825-5p mediates cleavage of endogenous *AT5G38850* transcripts in Arabidopsis. Thus, we named *AT5G38850 MIST1* for miRNA-silenced TNL-1, as the first TNL for which miRNA regulation has been experimentally demonstrated in Arabidopsis.

### miR825-5p triggers phasiRNA production from target *MIST1* transcripts

**Fig. 5.**
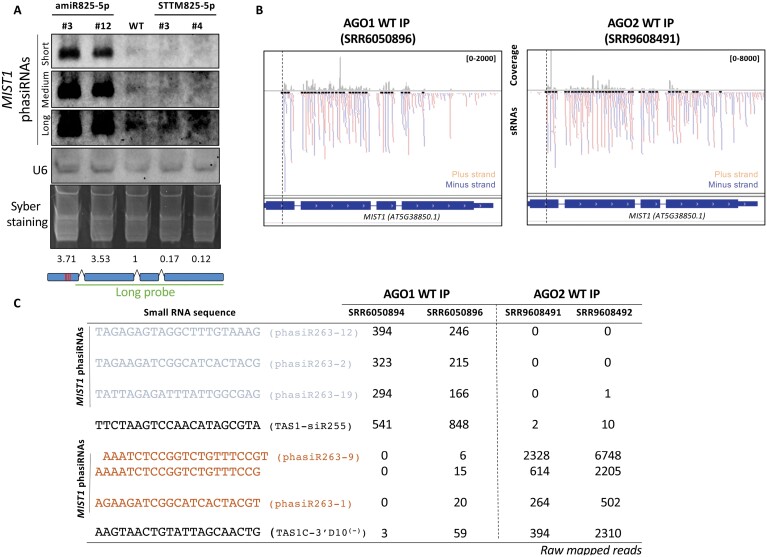
*MIST1*-derived phasiRNAs accumulate according to miR825-5p levels and are loaded onto AGO1 or AGO2 complexes. (A) Small RNA northern blot analysis of MIST1-derived phasiRNAs in amiR825-5p and STTM825-5p transgenic plants compared with the wild type (WT). U6 and Syber staining are used to control for loading. Three different exposures are shown. Normalized values in relation to WT levels calculated for each lane using ImageJ are shown below the corresponding lane. The long probe corresponding to the length of the gene used is also shown. Similar results were obtained in two biological replicates. (B) IGV screenshot showing production of sRNAs from the *MIST1* genomic region that are pulled down with AGO1 and AGO2 complexes. Corresponding libraries are indicated. (C) The table shows raw mapped reads for *MIST1*-derived most abundant phasiRNAs in AGO1/AGO2 pull-down experiments. Corresponding libraries are indicated. Well-established secondary tasiRNAs generated from *TAS1* genes are included as references.

Some reports have suggested that miR825-5p may trigger production of sRNAs from *MIST1* (Chen *et al.*, 2010; Niu *et al.*, 2016). In accordance, sRNAs mapping to *MIST1* have been found in several public databases (Howell *et al.*, 2007; Chen *et al.*, 2010; Niu *et al.*, 2016). Interestingly, the number of 21 nt sRNAs that accumulate from *MIST1* is the highest detected for an NLR gene in Arabidopsis, almost 6-fold higher than the number generated from the miR472 top CNL target (Supplementary Fig. S4). Also, *MIST1* is the only miR825-5p target from which 21 nt sRNAs accumulate significantly. Indeed, except for those triggered by miR472 from most of its targets, other NLRs mostly accumulate 24 nt, instead of 21 nt sRNAs, not consistent with miRNA/DCL4/RDR6-mediated phasiRNA production (Supplementary Fig. S5). The latter include *AT1G63750*, a miR825-5p target proposed as a phasiRNA-producing locus (Cai *et al.*, 2018) (Supplementary Fig. S5). Thus, miR825-5p differs notably from most reported 22 nt NLR-targeting miRNAs that generate phasiRNAs from most of their targets (Zhai *et al.*, 2011; Boccara *et al.*, 2014; Deng *et al.*, 2018; Canto-Pastor *et al.*, 2019). Analyses of the structure and characteristics of miR825-5p and *MIST1*-derived sRNAs support miR825-5p as the phasiRNA trigger, since: (i) miR825-5p is predicted to arise from an asymmetric fold-back precursor containing asymmetric bulges (Supplementary Fig. S6A), often linked to phasiRNA production (Chen *et al.*, 2010; Cuperus *et al.*, 2010; Manavella *et al.*, 2012); (ii) the first sRNA that significantly accumulates from the positive strand maps between positions 10 and 11 after the cleavage site (Supplementary Fig. S6B–D), as described for 22 nt-triggered phasiRNA production; (iii) sRNAs accumulate in a phased manner from the cleavage site, fitting with DCL4-mediated production (Supplementary Fig. S6E); (iv) phasiRNAs are typically produced from the target fragment displaying the least stable base pairing to the miRNA (Branscheid *et al.*, 2015), in this case the 3′ target fragment (Supplementary Fig. S6F), fitting with *MIST1*-derived sRNAs accumulating from the 3′ side of the miR825-5p target sequence; and; in addition, (v) analysis of *MIST1*-derived sRNA levels in adult leaves of *dcl2/4* and *rdr6* Arabidopsis mutants show that RDR6 and DCL4 are required for their accumulation, thus linking it to the canonical biogenesis pathway (Supplementary Fig. S6G). Finally, sRNA northern blot analysis of transgenic lines displaying elevated or decreased levels of mature miR825-5p (amiR825-5p and STTM825-5p, respectively) show that accumulation of phasiRNAs from *MIST1* (Fig. 5A) directly correlates with the levels of mature miR825-5p (Fig. 5A). The level of correlation between miRNA trigger and phasiRNA is similar to those reported for other 22 nt miRNAs (Cui *et al.*, 2020).

### miR825-5p-triggered phasiRNAs can act *in trans* to silence gene expression

**Fig. 6.**
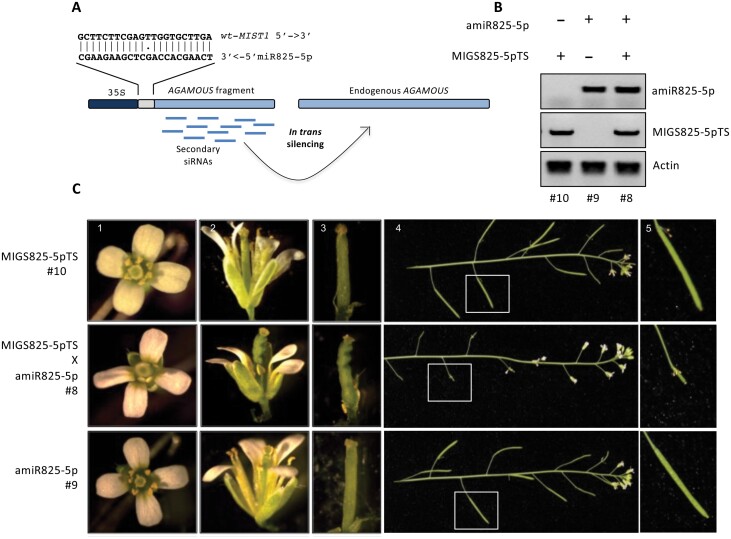
miR825-5p triggers transitivity at the *MIST1* target site. (A) Experimental design for MIGSS825-5p-TS. The miR825-5p target site from *MIST1* is fused to a 500 bp fragment of the *AGAMOUS* gene and expression of the construct is driven by a 35S constitutive promoter. Recognition by the RISC–miR825-5p complex is expected to trigger production of phasiRNAs from the AGAMOUS fragment, which are then expected to silence the endogenous *AGAMOUS* gene *in trans*. (B) DNA genotyping of plants with the amiR825-5p (#9), MIGS825-5pTS (#10), or both constructs (#8). (C) Flower and silique phenotypes for the different genotypes.

We analyzed raw data from available databases to conclude that *MIST1-*derived phasiRNAs are loaded onto AGO1 and AGO2 complexes (Fig. 5B, C), supporting their involvement in silencing. To demonstrate that the miR825-5p *MIST1* target site can trigger functional phasiRNAs capable of *trans*-gene silencing, we used miRNA-induced gene silencing (MIGS) technology (Fig. 6) (Felippes *et al.*, 2012) to generate transgenic plants expressing a gene fusion between the miR825-5p *MIST1* target sequence and an Arabidopsis *AGAMOUS* gene fragment (CaMV 35S::MIGS825-5pTS; Fig. 6A). miR825-5p triggering sRNA production at the *MIST1* target site would lead to the generation of phasiRNAs from the *AGAMOUS* fragment of the transcript that would silence expression of endogenous *AGAMOUS*, causing typical flower phenotypes associated with the lack of *AGAMOUS* function (Bowman *et al.*, 1989; Felippes *et al.*, 2012). Transgenic plants expressing MIGS825-5pTS displayed no apparent flower phenotype (Fig. 6B, C), probaby because the levels of mature miR825-5p in flowers are not sufficient to silence the highly expressed *AGAMOUS* gene (Drews *et al.*, 1991). Related to this, levels of 21 nt miR825-3p are significantly lower in flowers than in leaves (Vazquez *et al.*, 2008). Descendants from a cross between the lines carrying the *AGAMOUS* sensor system and plants expressing miR825-5p, carrying both constructs (MIGS825-5pTS and amiR825-5p), displayed flower phenotypes typically associated with mild to moderate silencing of the *AGAMOUS* gene (Wollmann *et al.*, 2010), including a distorted pistil, lack of maturation of the stamens, and infertility (Fig. 6B, C). Control plants expressing only amiR825-5p displayed wild-type phenotypes (Fig. 6B, C). These results support that miR825-5p triggers *in trans* silencing of endogenous *AGAMOUS* when acting at the target site of *MIST1.*

To investigate whether endogenous *MIST1*-derived phasiRNAs mediate gene silencing, we followed the approach used in legumes and *Solanaceae* (Zhai *et al.*, 2011; Deng *et al.*, 2018), using raw data from PARE libraries of Arabidopsis to look for degradome tags supporting phasiRNA targeting of TNL transcripts (Table 1). Applying the criteria used in *Solanaceae* (precise mapping of reads to predicted the cleavage position within the phasiRNA sequence) (Deng *et al.*, 2018) to be fulfilled in at least two independent libraries, and after filtering for those with a peak category 0–2, we found 24 degradome tags, corresponding to as many phasiRNAs, supporting *cis*-cleavage of *MIST1* transcripts, and 23 tags mapping to 15 different TNL genes (Table 1; Supplementary Fig. S7). These included the miR825-5p predicted primary target *AT1G63750*, for which degradome tags mapping to its cleavage site were also found. Using the more astringent criteria used in legumes (Zhai *et al.*, 2011) (Ws/Wl ratio ≥0.5), we found six tags within *MIST1*, and five within as many secondary TNL genes (Table 1).

**Table 1.** Degradome analysis using CleaveLand4

PhasiRname	Target	Cleavage position	Allen score	Library (Ws/Wl)^*a*,*b*^
phasiR263-10	AT1G56520.3	579	4	SRR1171802 (0.25), SRR1171803 (0.22)
phasiR263-13	AT1G58602.2	4337	3	SRR1171802 (0.31), SRR1171803 (0.26), SRR10322040 (0.20)
phasiR263-14	AT1G58807.1	3135	3,5	SRR1171802 (0.26), SRR1171803 (0.23), SRR1171804 (0.30), SRR10322040 (0.21)
phasiR263-14	AT1G58848.1	3333	3,5	SRR1171802 (0.27), SRR1171803 (0.23), SRR1171804 (0.30), SRR10322040 (0.12)
phasiR263-14	AT1G59124.2	3135	3,5	SRR1171802 (0.26), SRR1171803 (0.23), SRR1171804 (0.30), SRR10322040 (0.21)
phasiR263-15	AT1G63350.1	1653	3,5	**SRR1171802 (0.57)**, SRR1171803 (0.31)
phasiR263-38	AT1G63750.1	3127	5	SRR1171802 (0.41), SRR1171803 (0.40)
phasiR263-31	AT1G63870.1	1967	2,5	SRR1171802 (0.28), SRR1171803 (0.46)
phasiR263-6	AT1G63880.1	492	1	**SRR1171803 (0.65), SRR10322040 (0.57)**
phasiR263-44	AT1G63880.1	2672	3,5	SRR1171802 (0.20), SRR1171803 (0.23)
phasiR263-21	AT1G63880.1	1668	4	SRR1171802 (0.23), SRR1171803 (0.30)
phasiR263-20	AT1G63880.2	1632	3,5	SRR1171802 (0.23), SRR1171803 (0.30)
phasiR263-4	AT1G72910.1	628	4	SRR1171802 (0.25), SRR1171803 (0.18)
phasiR263-5	AT1G72910.1	631	5	SRR1171802 (0.24), SRR1171803 (0.16)
phasiR263-3	AT1G72910.1	626	5	SRR1171802 (0.16), SRR1171803 (0.19)
phasiR263-8	AT1G72940.1	637	4	SRR1171802 (0.054), SRR1171803 (0.11)
phasiR263-5	AT1G72940.1	600	5	SRR1171802 (0.10), SRR1171803 (0.12)
phasiR263-22	AT3G44630.2	3682	3,5	SRR1171802 (0.07), SRR1171803 (0.11)
phasiR263-26	AT4G14370.1	1626	4,5	**SRR1171802 (1), SRR1171803 (0.62)**
phasiR263-29	AT5G46260.1	805	4,5	SRR1171803 (0.48), SRR10322040 (0.40)
phasiR263-11	AT5G46260.1	787	3	SRR1171803 (0.22), **SRR1171804 (1)**
phasiR263-11	AT5G46490.1	714	3	SRR1171803 (0.23), **SRR1171804 (1)**
phasiR263-29	AT5G46490.8	660	4,5	SRR1171803 (0.48), SRR10322040 (0.40)
phasiR263-26	*MIST1*	1753	0	SRR1171802 (0.38), SRR1171804 (0.34)
phasiR263-17	*MIST1*	1188	0	SRR1171802 (0.34), **SRR1171804 (1)**
phasiR263-41	*MIST1*	2228	0	SRR1171802 (0.17), SRR1171803 (0.13)
phasiR263-7	*MIST1*	337	0	**SRR1171802 (0.71)**, SRR10322040 (0.36)
phasiR263-42	*MIST1*	2278	3	SRR1171802 (0.39), SRR1171803 (0.24)
phasiR263-25	*MIST1*	1713	0	SRR1171802 (0.15), SRR1171803 (0.26)
phasiR263-16	*MIST1*	1103	0	SRR1171803 (0.41), **SRR1171804 (1)**
phasiR263-18	*MIST1*	1281	0	SRR1171802 (0.26), SRR1171803 (0.15),
phasiR263-43	*MIST1*	2331	0	SRR1171802 (0.35), SRR1171803 (0.26), **SRR1171804 (0.50)**
phasiR263-32	*MIST1*	1946	0	SRR1171802 (0.36), SRR1171803 (0.26), SRR10322040 (0.22)
phasiR263-28	*MIST1*	1837	0	**SRR1171802 (0.80), SRR1171803 (0.84)**
phasiR263-23	*MIST1*	1535	0	SRR1171802 (0.35), SRR1171803 (0.34)
phasiR263-30	*MIST1*	1917	0	SRR1171802 (0.39), SRR1171803 (0.38)
phasiR263-24	*MIST1*	1580	0	SRR1171802 (0.27), SRR1171803 (0.25)
phasiR263-37	*MIST1*	2193	0	SRR1171802 (0.27), SRR1171803 (0.17)
phasiR263-35	*MIST1*	2079	0	SRR1171802 (0.16), SRR1171803 (0.20)
phasiR263-36	*MIST1*	2162	0	SRR1171802 (0.33), SRR1171803 (0.35)
phasiR263-39	*MIST1*	2223	0	SRR1171802 (0.23), SRR1171803 (0.24)
phasiR263-45	*MIST1*	2742	0	SRR1171803 (0.21), SRR1171804 (0.41)
phasiR263-33	*MIST1*	1947	0	SRR1171802 (0.35), SRR1171803 (0.30)
phasiR263-27	*MIST1*	1810	0	SRR1171802 (0.36), SRR1171803 (0.21)
phasiR263-40	*MIST1*	2225	0	SRR1171802 (0.20), SRR1171803 (0.24)
phasiR263-34	*MIST1*	2005	0	SRR1171803 (0.34), **SRR1171804 (1)**

a Ratio of the number of reads in the small window Ws (5 ±2 nt) to the number of reads in the large window Wl (31±15 nt) (Zhai *et al.*, 2011).

b Bold highlights libraries displaying a Ws/Wl ratio ≥0.5.

### The *MIR825* promoter is down-regulated in response to perception of PAMPs

*MIR825*-derived miRNAs have been described as differentially accumulated in plants infected by *P. syringae*, when these had been pre-treated with *B. cereus* (Niu *et al.*, 2016). On the other hand, separate studies reported that levels of *MIR825*-derived miR825-3p decrease in response to *P. syringae* alone (Fahlgren *et al.*, 2007; Zhang *et al.*, 2010; Niu *et al.*, 2016). To clarify this issue, we investigated expression of *MIR825* in more detail. Inoculation with *P. syringae* caused a 70% decrease in pri-miR825 accumulation at 3 hours post-inoculation (hpi; Supplementary Fig. S8A). A similar reduction was observed after treatment with flg22 (Supplementary Fig. S8B), suggesting that flagellin perception is involved in triggering *MIR825* down-regulation. However, down-regulation is not specific to flagellin perception since: (i) Col-0 *fls2* mutant plants (unable to perceive flagellin) display a similar decrease in response to *P. syringae* (Supplementary Fig. S8C) and (ii) down-regulation can also be triggered by the fungal PAMP chitin (Supplementary Fig. S8D), the latter in keeping with a recent report (Nie *et al.*, 2019). Since type III effectors with silencing-suppressing activities have been proposed to down-regulate miR482 levels in tomato, we tested the response to an *hrcV* mutant, defective in type III secretion. pri-miR825 accumulation was significantly reduced upon treatment with this mutant (Supplementary Fig. S8E), although to a lower extent than with the wild type.

**Fig. 7.**
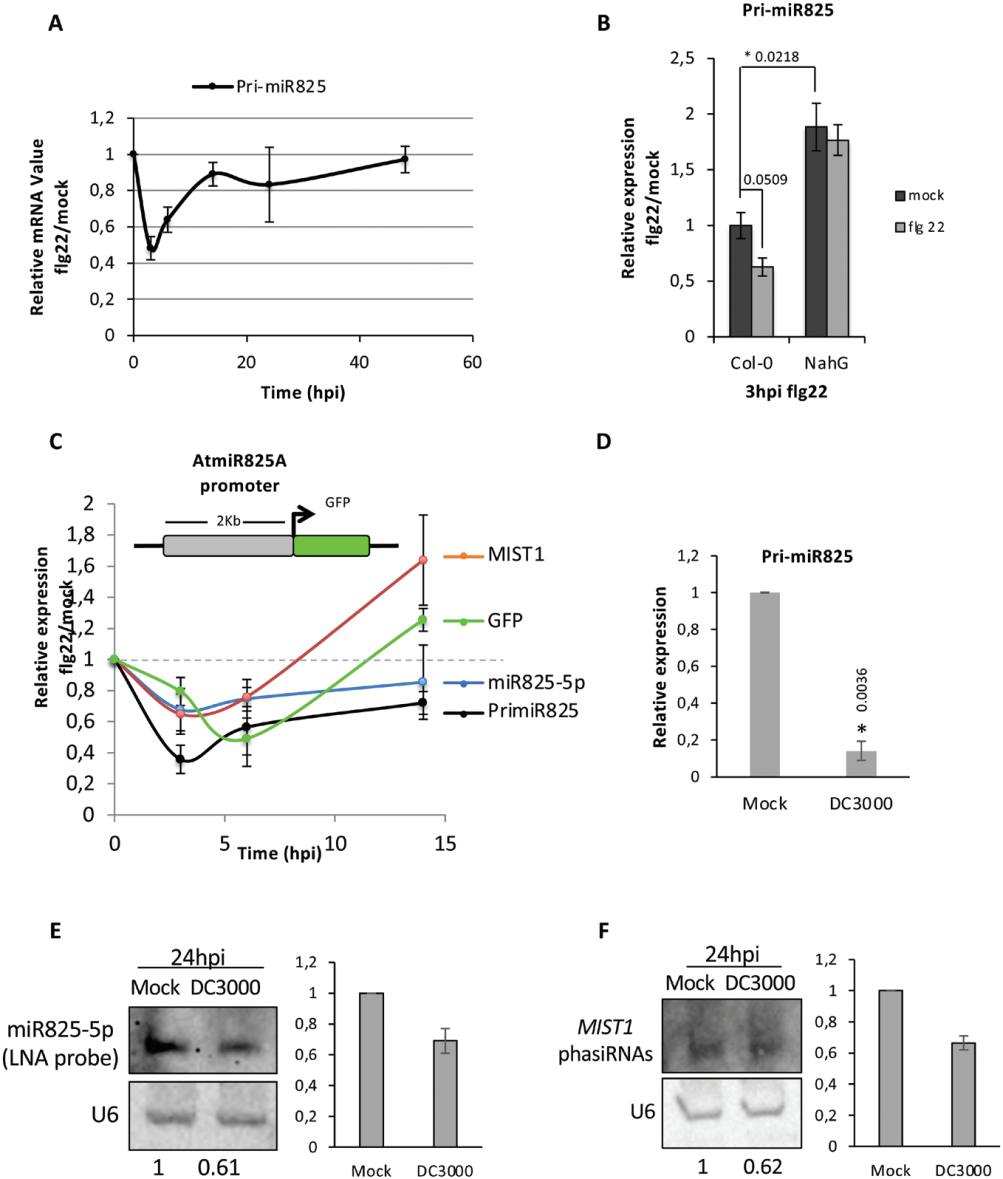
Levels of pri-miR825 and miR825-5p are down-regulated upon PAMP perception. (A) Time-course experiment using RT–qPCR to follow accumulation of pri-miR825 transcripts after flg22 treatment. Error bars correspond to the SE. (B) RT–qPCR of pri-miR825 transcripts in wild-type and transgenic *NahG* plants 3 h post-mock or flg22 treatment. Asterisks indicate that results are significantly different from each other, as established by a Student’s *t*-test (*P*<0.05). Error bars correspond to the SE. Numbers above bars indicate the *P*-value. (C) Time-course experiment after flg22 treatment of a pMIR825*::GFP* transgenic line, using RT–qPCR to follow accumulation of endogenous pri-miR825 transcripts, miR825-5p levels, and *MIST1* transcripts, as well as transcripts from the transgene formed by fusion of the AtMIR825 promoter to the GFP-coding sequence. Error bars correspond to the SE. (D) RT–qPCR of pri-miR825 transcripts in wild-type plants 24 hpi with a mock solution or a 5×10^7^ cfu ml^–1^ suspension of *P. syringae* DC3000. An asterisk indicates that results are significantly different from each other, as established by a Student’s *t*-test (*P*<0.05). Error bars correspond to the SE. The number above indicates the *P*-value. Similar results were obtained in two biological replicates. (E) Northern blot analysis of the levels of miR825-5p in RNA samples taken from adult leaves 24 h post-inoculation with a 5×10^4^ cfu ml^–1^ suspension of either Pto DC3000 (Pto) or the inoculating solution (mock). The accompanying graph shows the band intensity quantified with ImageJ in relation to mock. Similar results were obtained in two biological replicates. (F) Northern blot analysis of the levels of sRNAs produced from the *MIST1* transcript in RNA samples taken from adult leaves 24 h post-inoculation with either Pto DC3000 (5×10^4^ cfu ml^–1^ suspension) or the inoculating solution (mock). The accompanying graph shows the band intensity quantified with ImageJ in relation to mock. Similar results were obtained in two biological replicates.

A time-course experiment carried out following flg22 treatment (Fig. 7A) showed a drastic drop in pri-miR825 accumulation 3 h after treatment, followed by a slow recovery up to 48 h. Flg22-mediated down-regulation of pri-miR825 levels is lost in plants carrying the *NahG* transgene (Fig. 7B) that accumulate very low concentrations of SA and present enhanced susceptibility to *P. syringae* (Delaney *et al.*, 1994), suggesting that down-regulation of *MIR825* expression is SA dependent. Supporting this notion, untreated *NahG* plants presented significantly higher levels of pri-miR825 than wild-type untreated plants (Fig. 7B).

To determine if down-regulation of *MIR825* upon PAMP perception takes place at the transcriptional level, we generated Arabidopsis lines expressing the *GFP* gene under the control of the *MIR825* promoter (Fig. 7C). Western blot analysis of GFP levels in independent transgenic lines confirmed GFP protein accumulation in adult leaves (Supplementary Fig. S9). Treatment of these plants with flg22 caused a similar dynamic for GFP mRNA and pri-miR825 accumulation (Fig. 7C), with a slight delay for GFP mRNA accumulation. This delay might be due to DCL1 processing of pri-miR825, which is likely to accelerate precursor reduction. *MIST1* transcripts, which followed a downward trend for the first few hours, showed a clear and sustained increase by 14 h (Fig. 7C). Interestingly, although levels of pri-miR825 had recovered considerably 24 h after treatment with flagellin, these were still significantly lower 24 hpi with *P. syringae*, perhaps due to sustained exposure to flagellin and/or to additional PAMPs (Fig. 7D). Levels of miR825-5p reproducibly displayed a decrease 24 hpi, although milder that seen for pri-miR825 levels (Fig. 7E), in keeping with the results from the time-course experiment (Fig. 7C). A similar trend could be observed for phasiRNAs generated from *MIST1* transcripts at both time points (Fig. 7F).

## Discussion

Numerous examples of miRNA-mediated siRNA-generating loci have been reported in *Solaneaceae* and legume species (Fei *et al.*, 2013). Remarkably fewer NLR loci displaying such regulation have been described in *Brassicaceae*. miR472 targets the sequence encoding the P-loop of several CNL genes in Arabidopsis, triggering the generation of a small number of siRNAs with *cis*-silencing activity (Boccara *et al.*, 2014). Also, changes in the levels of the two miRNAs generated from *MIR825* had been reported to negatively correlate with transcript levels for several genes, including *AT5G38850* (Niu *et al.*, 2016; Nie *et al.*, 2019). In this work, we provide full experimental evidence for the intuitively reasonable conclusion that *AT5G38850* is a direct target for endonucleolytic cleavage by the 22 nt miRNA generated from *MIR825*—miR825-5p—previously known as miR825*. This conclusion is supported by the following: (i) altered levels of miR825-5p negatively correlate with *AT5G38850* mRNA levels in transgenic plants (Supplementary Fig. S2); (ii) transient co-expression with miR825-5p in *N. benthamiana* of reporter constructs carrying the *AT5G38850* miR825-5p target site fused to *GFP*, or a mutated version lacking miR825-5p complementarity, shows that miR825-5p silences *GFP* in a target site complementarity-dependent manner (Fig. 4B, C); and (iii) degradome analysis of PARE libraries shows significantly accumulated degradome tags mapping precisely to the miR825-5p cleavage site on *AT5G38850* (Fig. 4D). On this basis, we have named *AT5G38850* as *MIST1* for miRNA silenced TNL1, as the first TNL gene for which miRNA silencing has been experimentally demonstrated in Arabidopsis.

miR825-5p targets a sequence coding for a highly conserved motif, TIR2 (Supplementary Fig. S10), adjacent to the catalytic residue for NAD^+^-cleaving enzymatic activity, essential for TNL immune function (Wan *et al.*, 2019). Targeting of conserved sequences of functional relevance provides an evolutionary link between protein function and miRNA-mediated regulation. Examples of such links are found in *Medicago sativa* where miRNA regulation of CNLs, and a few TNLs, is performed through targeting of the sequences coding for the P-loop (Shivaprasad *et al.*, 2012) or the Kinase-2 motif of the CC domain of CNLs (Zhai *et al.*, 2011). At least two miRNAs have been shown to target the conserved TIR1 motif (Zhai *et al*., 2011; Deng *et al.*, 2018); however, no biochemical activity has been associated with this motif. Although the TIR2 motif has no demonstrated function, its position immediately adjacent to the catalytic residue for the NAD^+^-cleaving enzymatic activity, essential for TNL function (Wan *et al.*, 2019) makes any changes on this motif more likely to affect enzymatic activity.

The 22 nt miRNAs trigger RDR6-dependent production of secondary phasiRNA from many NLRs. In Arabidopsis, miR825-5p has been proposed to trigger siRNA production from two TNLs, *AT5G38850* (Howell *et al.*, 2007; Chen *et al.*, 2010; Niu *et al.*, 2016; Cai *et al.*, 2018) (*MIST1*) and *AT1G63750* (Cai *et al.*, 2018). Our sRNA northern assays (Fig. 5) and bioinformatic analysis support *MIST1*, but not *AT1G63750* (Supplementary Figs S5, S6), as an miR825-5p-triggered phasiRNA-producing locus. miR825-5p triggers the production of a remarkably large number of phasiRNAs from *MIST1* transcripts, the highest for an NLR gene in Arabidopsis (Supplementary Fig. S4). This substantial number, within the range of the numbers of tasiRNAs generated from non-coding loci (50% of *TAS1A*, 30% of *TA*S3), contrasts with the limited number (almost 6-fold less; Supplementary Fig. S4) of phasiRNAs generated by miR472 from the top CNL target (Boccara *et al.*, 2014). miR825-5p regulation of TNLs differs further from miR472 regulation of CNLs by the fact that whereas miR472 triggers siRNA production from most of its targets, miR825-5p only triggers significant 21 nt production from *MIST1*.

Regarding the function of miR825-5p-triggered *MIST1*-generated phasiRNAs, we show that: (i) phasiRNAs generated from *MIST1* are loaded onto AGO1/AGO2 complexes (Fig. 5); (ii) miR825-5p triggers, from its target site on *MIST1*, silencing of an *AGAMOUS*-based transitivity reporter; and (iii) degradome analysis of PARE libraries reveals the presence of tags that by fulfilling the restrictive criteria established in legumes (Zhai *et al.*, 2011) and *Solanaceae* (Deng *et al.*, 2018), match precisely to the predicted cleavage site of several *MIST1*-derived phasiRNAs on a number of gene targets (Table 1). These results support that miR825-5p-triggered *MIST1*-derived phasiRNAs establish a second layer of regulation by endonucleolytic cleavage on TNL genes, reinforcing silencing of primary target *MIST1* and perhaps providing feedback regulation for subsequent phasiRNA production, and extending the network to include secondary targets (Table 1). Two circumstances could prevent a phasiRNA target from being identified by degradome analysis: (i) *trans*-silencing by siRNAs being performed by translational inhibition as has been proposed (Deng *et al.*, 2018; Canto-Pastor *et al.*, 2019); and (ii) not being expressed under basal conditions, as is the case for many NLR genes. Thus, the regulatory network established by *MIST1*-generated phasiRNAs may be larger than that revealed by our analysis of PARE libraries. To evaluate the potential reach of such an extended network, we searched for targets for the top five most abundant *MIST1*-derived phasiRNAs among those loaded onto AGO1 and/or AGO2 (Supplementary Fig. S10; Supplementary Table S11). This analysis points to phasiR263-9 as a potential secondary hub for TNL regulation, since it displays distinctly larger numbers of both reads associated with AGO2 complexes (Fig. 5) and predicted targets (Supplementary Fig. S10). phasiR263-9 targets TIR3, another highly conserved motif within the TIR domain (Meyers *et al.*, 1999).

miR472- and miR482/2118-mediated NLR silencing is lifted during the onset of immunity (Shivaprasad *et al.*, 2012; Boccara *et al.*, 2014; Su *et al.*, 2018) when targeted NLR genes become transcriptionally active. Here, we show that *MIR825* is transcriptionally down-regulated upon contact with pathogenic *P. syringae* (Fig. 7). *MIR825* down-regulation upon *P. syringae* infection can be fully recapitulated by treatment with flagellin or the fungal PAMP chitin, and partially by inoculation with the type III mutant *hrcV* (Supplementary Fig. S8). Thus, our results support a link between PAMP perception and *MIR825* down-regulation. We also found that *MIR825* down-regulation is SA dependent. SA dependency has also been shown for the impact of RDR6 on basal defenses in Arabidopsis (Boccara *et al.*, 2014). Prior to our study, *MIR825*-derived miRNAs were described to differentially accumulate in plants undergoing ISR and subsequently infected by *P. syringae* or *B. cinerea*, although accumulation in response to pathogen infection alone in this study was found not to be significant (Niu *et al.*, 2016; Nie *et al.*, 2019). However, several studies reported that miR825-3p decreases in response to pathogenic or type III secretion-defective *P. syringae* (Fahlgren *et al.*, 2007; Zhang *et al.*, 2010), in keeping with our results. These discrepancies may be due to differences in experimental setting and timing. We also show that *MIR825* down-regulation is accompanied by a decrease in miR825-5p accumulation and leads to the accumulation of *MIST1* mRNA and to a mild decrease in *MIST1*-derived phasiRNAs. Differences in the latter are small but, since phasiRNA silencing involves a number of targets, the impact on immunity may be additive, as has been previously proposed for a similar case (Shivaprasad *et al.*, 2012).

Here we show that full PTI requires lifting of miR825-5p silencing of TNLs, through either flg22 treatment or *P. syringae* infection. Recent reports have shown that NLR activation augments gene expression and potentiates PRR-mediated responses (Adachi *et al.*, 2015; Ngou *et al*., 2020; Yuan *et al.*, 2021). Such a regulatory set-up, where NLR activation contributes to full PTI, might allow the plant to overcome PTI suppression imposed by the activity of pathogen effectors, such as *P. syringae* type III effectors, and provides the potential molecular mechanism behind our results. Nonetheless, since miR825-5p-targeted genes encode uncharacterized NLRs, it seems plausible that miR825-5p might also regulate ETI. On this note, *AT5G18360*, a putative miR825-5p target, has been recently described to be involved in triggering HopB-mediated immunity (Laflamme *et al.*, 2020). Our results support that miR825-5p silences TNL expression in basal, unchallenged conditions, and is potentially involved in lowering the fitness cost of surveillance, which results from expressing NLR genes in the absence of pathogen attack (Moreno-Gámez *et al.*, 2013). In addition, miR825-5p could also be involved in buffering NLR transcript levels, dampening dramatic changes through phasiRNA-mediated feedback regulation (Fei *et al.*, 2013). Recent reports have shown that, unlike CNLs, TNLs do not activate the hypersensitive response in transgenic plants expressing the corresponding effectors unless flg22 is provided (Lu and Tsuda, 2020; Ngou *et al*., 2020; Yuan *et al.*, 2021). miR825-5p dampening of TNL expression being lifted by flg22 could provide a molecular mechanism for such a dependency. This report provides an additional molecular mechanism required for full expression of PTI that, through down-regulation of sRNA silencing, links PRR perception to TNL expression.

## Supplementary data

The following supplementary data are available at *JXB* online.

Fig. S1. Target analysis revealed miR825-5p as a central hub for TNL gene regulation.

Fig. S2. Controls for transgenic plants described in Figs 2 and 3.

Fig. S3. T-plots showing degradome tags for miRNA targets.

Fig. S4. TNL-encoding *AT5G38850* gene accumulates more sRNAs than any other NLR in Arabidopsis.

Fig. S5. siRNA production from different NLR genes.

Fig. S6. miRNA825-5p is a trigger for phasiRNAs production from *MIST1* transcripts.

Fig. S7. Regulatory network based on degradome data for miR825-5p and *MIST1*-derived miR825-5p-triggered phasiRNAs.

Fig. S8. pri-miR825 is down-regulated by PAMPs.

Fig. S9. *At*miR825A promoter is active in adult leaves.

Fig. S10. miR825-5p predicted regulatory network.

Table S1. Primers used in this work.

Table S2. ACT2 expression relative to UBQ5 in miR825-5p altered genotypes and statistical analysis.

Table S3. ACT2 expression relative to UBQ5 after flg22 treatment and statistical analysis.

Table S4. Antibodies used in this work.

Table S5. Strains used in this work.

Table S6. Plasmids used in this work.

Table S7. Transgenic lines generated in this work.

Table S8. Software and bioinformatic methods used in this work.

Table S9. Libraries used in this work.

Table S10. Extended list of targets for miR825-5p and 3p using two different prediction software programs.

Table S11. List of predicted targets of miR825-5p/MIST1/phasiTNLs.

erab354_suppl_Supplementary_Figures_S1-S10_Tables_S1-S9

erab354_suppl_Supplementary_Table_10-S11

## Data Availability

The data that support the findings of this study are available within the paper and its supplementary data.

## Abbreviations

CC: coiled-coil
CNL: CC-NLR
ETI: effector-triggered immunity
NLR: NOD-like receptor
PTI: PAMP-triggered immunity
ISR: induced systemic resistance
TIR: Toll/interleukin-1 receptor
TNL: TIR-NLR.
