# Supplementary material for: miR825-5p targets the TIR-NBS-LRR gene MIST1 and down-regulates basal immunity against Pseudomonas syringae in Arabidopsis
Source: J Exp Bot. 2021 Jul 30;72(20):7316–34. doi: 10.1093/jxb/erab354 (PMC8547151; doi:10.1093/jxb/erab354)
Supplement: erab354_suppl_Supplementary_Figures_S1-S10_Tables_S1-S9 [file erab354_suppl_supplementary_figures_s1-s10_tables_s1-s9.pdf]

Fig. S1

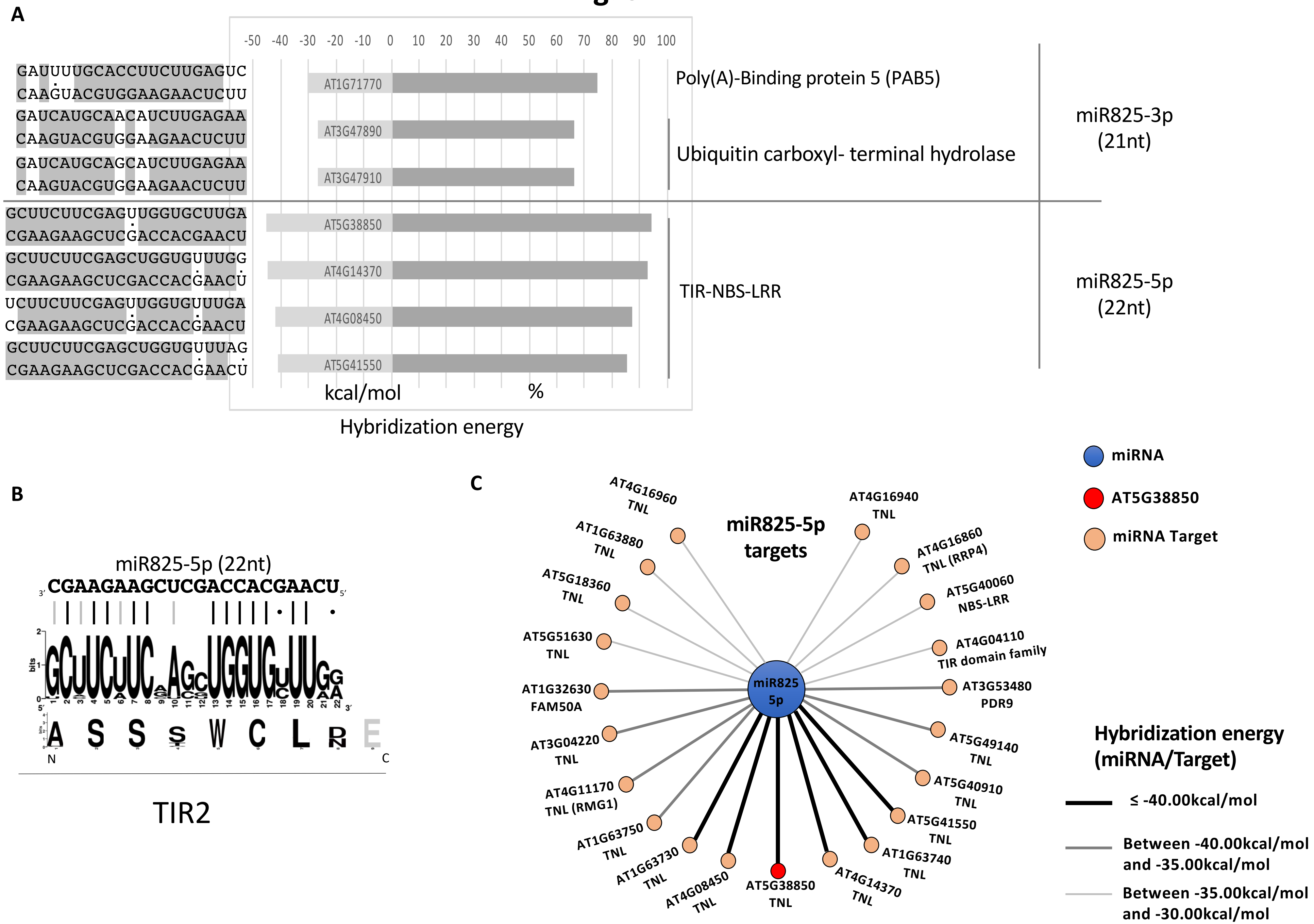
A
-50 -40 -30 -20 -10 0 10 20 30 40 50 60 70 80 90 100
GAUUUUGCACCUUCUUGAGUC
CAAGUACGUGGAAGAACUCUU
AT1G71770
Poly(A)-Binding protein 5 (PAB5)
GAUCAUGCAACAUCUUGAGAA
CAAGUACGUGGAAGAACUCUU
AT3G47890
GAUCAUGCAGCAUCUUGAGAA
CAAGUACGUGGAAGAACUCUU
AT3G47910
Ubiquitin carboxyl- terminal hydrolase
miR825-3p (21nt)
GCUUCUUCGAGUUGGUGCUUGA
CGAAGAAGCUCGACCACGAACU
AT5G38850
GCUUCUUCGAGCUGGUGUUUGG
CGAAGAAGCUCGACCACGAACU
AT4G14370
UCUUCUUCGAGUUGGUGUUUGA
CGAAGAAGCUCGACCACGAACU
AT4G08450
GCUUCUUCGAGCUGGUGUUUAG
CGAAGAAGCUCGACCACGAACU
AT5G41550
TIR-NBS-LRR
miR825-5p (22nt)
kcal/mol
%
Hybridization energy
B
miR825-5p (22nt)
3′ CGAAGAAGCUCGACCACGAACU 5′
bits
5′ 3′
A S S S W C L R E
N C
TIR2
C
miR825-5p targets
miR825 5p
AT4G16960 TNL
AT1G63880 TNL
AT5G18360 TNL
AT5G51630 TNL
AT1G32630 FAM50A
AT3G04220 TNL
AT4G11170 TNL (RMG1)
AT1G63750 TNL
AT1G63730 TNL
AT4G08450 TNL
AT5G38850 TNL
AT4G14370 TNL
AT1G63740 TNL
AT5G41550 TNL
AT5G40910 TNL
AT5G49140 TNL
AT3G53480 PDR9
AT4G04110 TIR domain family
AT5G40060 NBS-LRR
AT4G16860 TNL (RRP4)
AT4G16940 TNL
miRNA
AT5G38850
miRNA Target
Hybridization energy (miRNA/Target)
≤ -40.00kcal/mol
Between -40.00kcal/mol and -35.00kcal/mol
Between -35.00kcal/mol and -30.00kcal/mol

Fig. S1 Target analysis revealed miR825-5p as a central hub for TNL gene regulation. A We used WMD3 (Ossowski et al., 2008) and default parameters on Araport11 to predict targets for MIR825-encoded 21-nt (miR825-3p, formerly miR825) and 22-nt (miR825-5p; formerly miR825*). All three predicted targets for 21-nt miR825-3p are shown. Only the top four predicted targets for 22-nt miR825 are shown. Predicted targets for 22-nt miR825 are genes encoding Toll/interleukin-1 (TIR), nucleotide binding site (NBS) and leucine-rich repeat (LRR) containing proteins (TIR-NBS-LRR). **B** MiR825-5p sequence paired with the consensus for 18 TNLs (plus one TIR domain protein) putative targets from the *Arabidopsis* genome. The logo corresponding to the consensus protein sequence for miR825-5p target site is shown below (black lines indicates perfect pairing, grey lines perfect pairing with the most conserved nucleotide, and dots indicate variable region that allows pairing at the RNA level). **C** Primary network showing all 21 putative targets for miR825-5p as predicted using WMD3 and default parameters on Araport11. TNL is indicated for the 17 out of these 21 that are annotated as such. Two additional genes encode a truncated TIR-NBS-LRR and a TIR domain-carrying protein.

Fig. S2

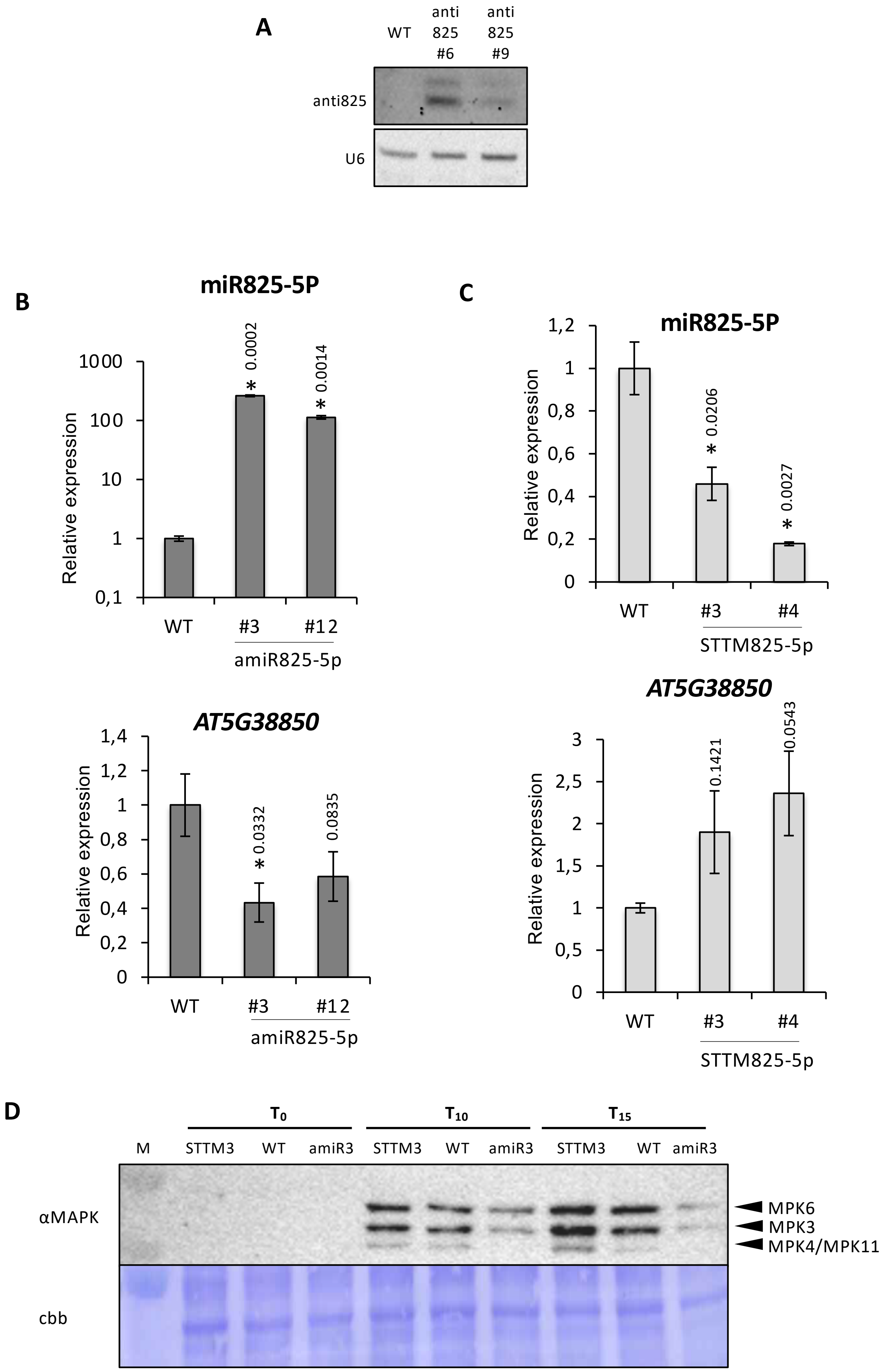
A
WT
anti 825 #6
anti 825 #9
anti825
U6
B
miR825-5P
Relative expression
1000
100
10
1
0,1
* 0.0002
* 0.0014
WT
#3
#12
amiR825-5p
C
miR825-5P
Relative expression
1,2
1
0,8
0,6
0,4
0,2
0
* 0.0206
* 0.0027
WT
#3
#4
STTM825-5p
AT5G38850
Relative expression
1,4
1,2
1
0,8
0,6
0,4
0,2
0
* 0.0332
0.0835
WT
#3
#12
amiR825-5p
AT5G38850
Relative expression
3
2,5
2
1,5
1
0,5
0
0.1421
0.0543
WT
#3
#4
STTM825-5p
D
T0
T10
T15
M
STTM3
WT
amiR3
STTM3
WT
amiR3
STTM3
WT
amiR3
αMAPK
cbb
MPK6
MPK3
MPK4/MPK11

Fig. S2 Controls for transgenic plants described in Fig. 2 and 3. A Small RNA Northern blot assay showing accumulation of artificial miRNA (amiR anti825; supplemental methods; **Fig. S2a**) (Schwab, 2006; Ossowski *et al.*, 2008), resulting from the modification of the precursor for miR319 to target and silence pri-miR825. **B** Accumulation of miR825-5p (upper panel) and *AT5G38850* (lower panel) in amiR825-5p transgenic plants. Asterisks indicate results are significantly different from WT plants, as established by a Student's t-test ($P<0.05$). Error bars correspond to standard error. **C** Accumulation of miR825-5p (upper panel) and *AT5G38850* (lower panel) in STTM825-5p transgenic plants. Asterisks indicate results are significantly different from WT plants, as established by a Student's t-test ($P<0.05$). Error bars correspond to standard error. **D** Western Blot analysis showing levels of phosphorylated mitogen-activated protein kinases (MPK3, MPK4, MPK6 and MPK11) after treatment with 100 nM flg22 of wild type (WT), an STTM line, and an amiR line at three different time points (0, 10 or 15 min post flg22 treatment). Similar results were obtained with additional lines. Membrane was stained with Coomasie as a loading reference.

Fig. S3

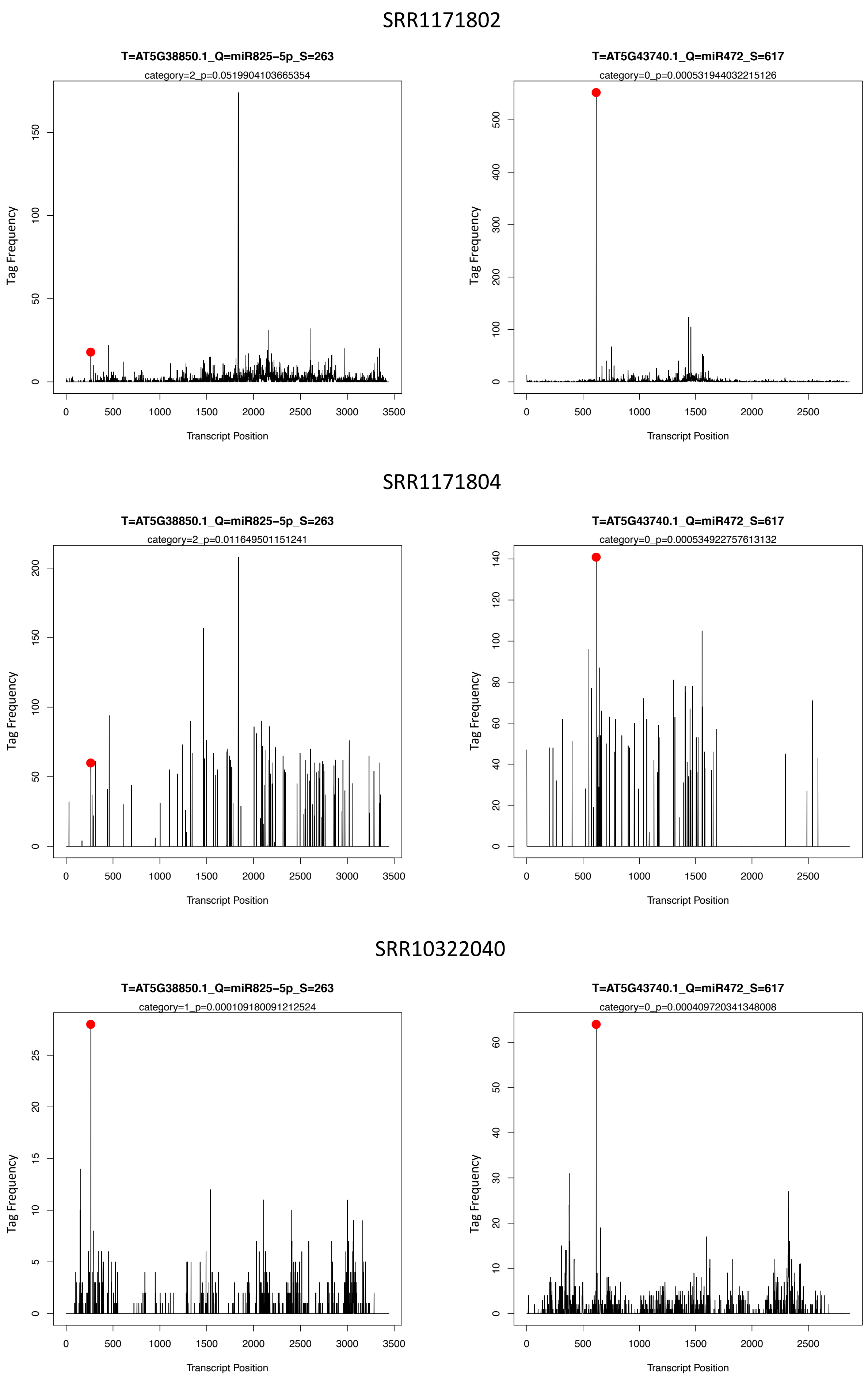

Fig. S3 T-plots showing degradome tags for miRNA targets. T-plots showing density of 5' position of degradome tags corresponding to *MIST1* (miR825-5p target) and *AT5G43740* (miR472 target) across different degradome libraries. The red dot indicates degradome tags starting at the predicted target site for miR825-5p or miR472 (slicing between10th-11th nucleotides at complementary target site relative to the miRNA). Category refers to PARE read abundance of that position (Addo-Quaye *et al.*, 2009). P-values are indicated.

Fig.S4

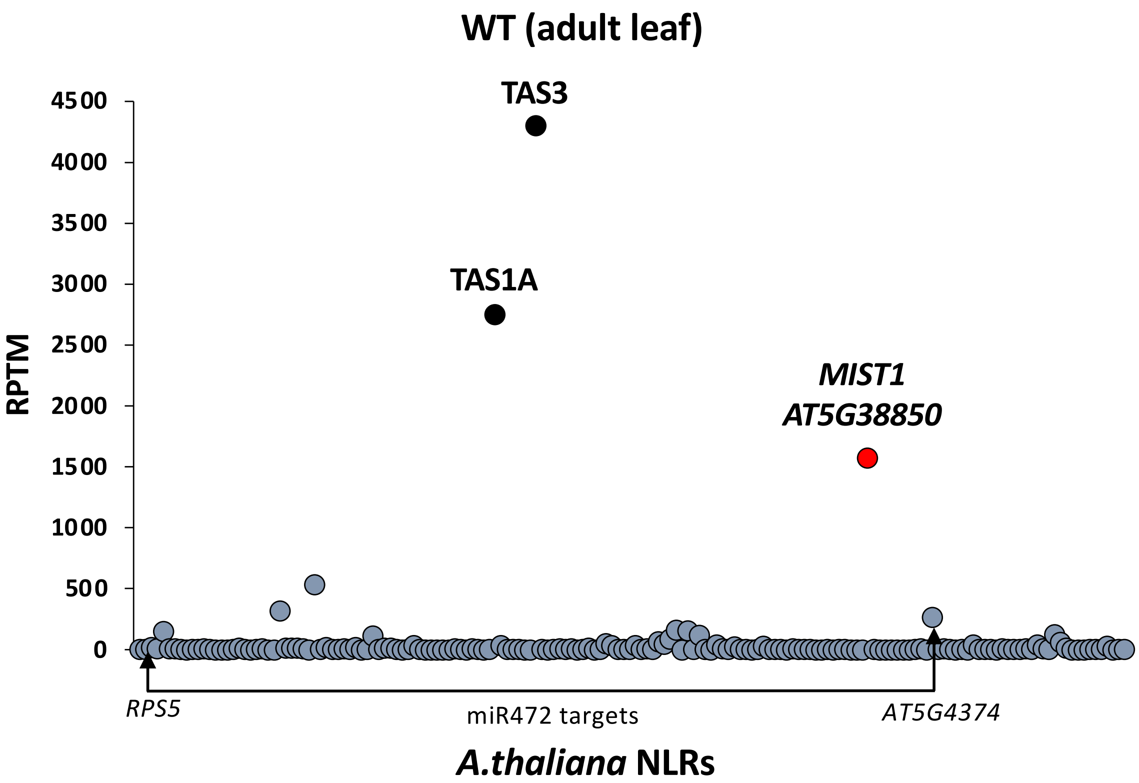

Fig. S4 TNL-encoding *AT5G38850* gene accumulates more sRNAs than any other NLR in Arabidopsis. Graph shows sRNA accumulation from all NLRs within the *Arabidopsis* genome (data obtained from NCBI: BioProject SRP097592, WT library). Graph displays number of sRNA (reads per 10 million small RNAs mapped, RPTM) accumulated from each NLR-encoding gene. Arrows point to two miR472 target CNL genes. Number of sRNA accumulated from *TAS1A* and *TAS3* are provided as reference.

Fig. S5

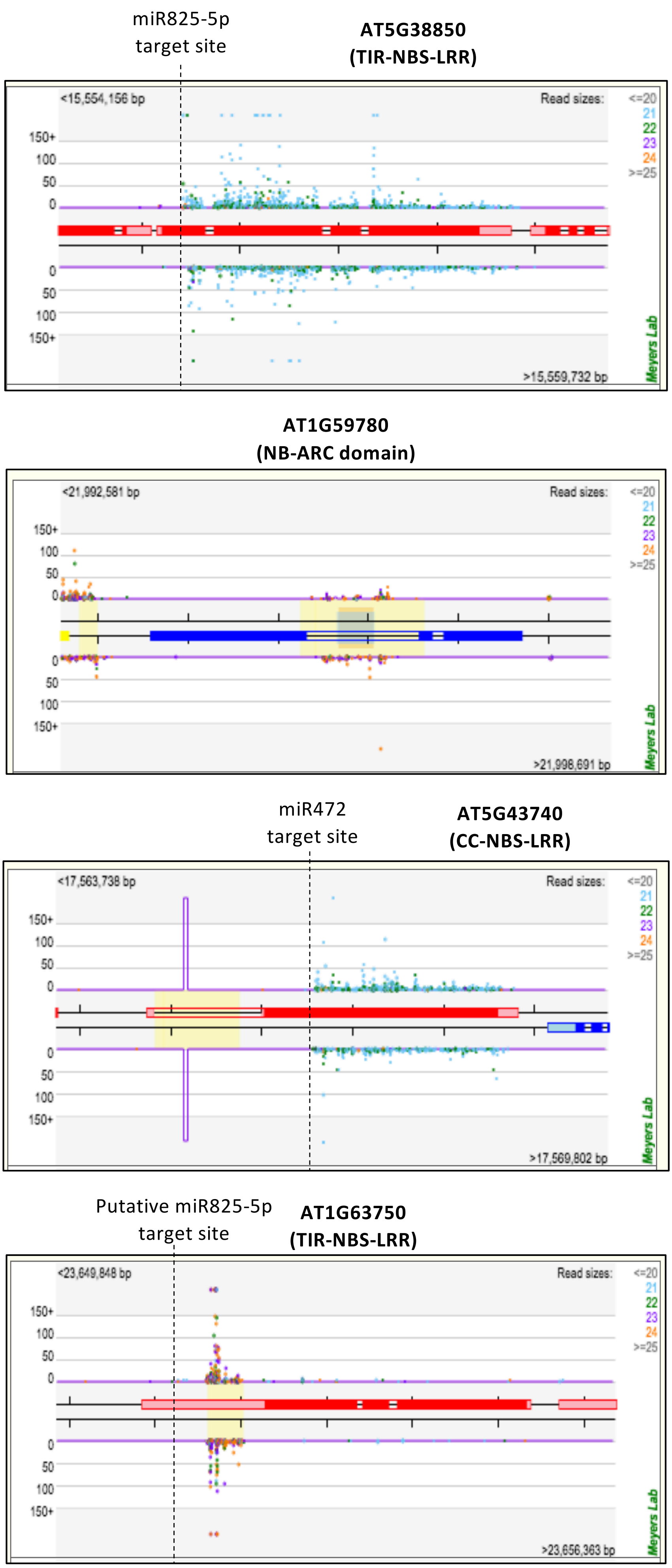
miR825-5p
target site
AT5G38850
(TIR-NBS-LRR)
<15,554,156 bp
Read sizes:
<=20
21
22
23
24
>=25
150+
100
50
0
>15,559,732 bp
Meyers Lab
AT1G59780
(NB-ARC domain)
<21,992,581 bp
>21,998,691 bp
miR472
target site
AT5G43740
(CC-NBS-LRR)
<17,563,738 bp
>17,569,802 bp
Putative miR825-5p
target site
AT1G63750
(TIR-NBS-LRR)
<23,649,848 bp
>23,656,363 bp

AT4G16920
(TIR-NBS-LRR)

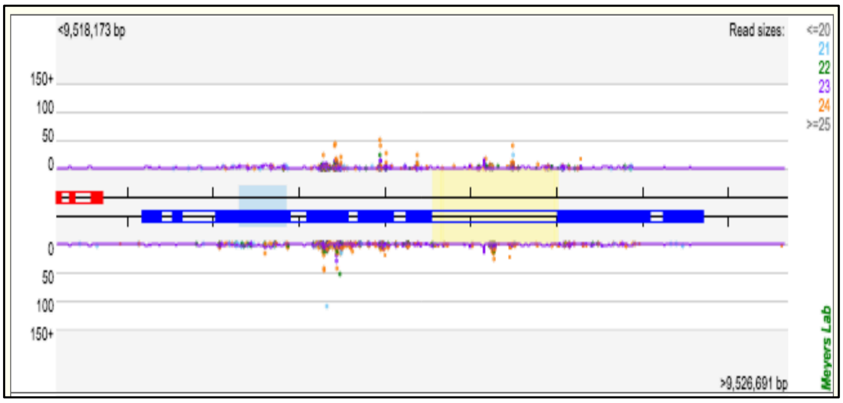

AT4G16950
(RPP5/TIR-NBS-LRR)

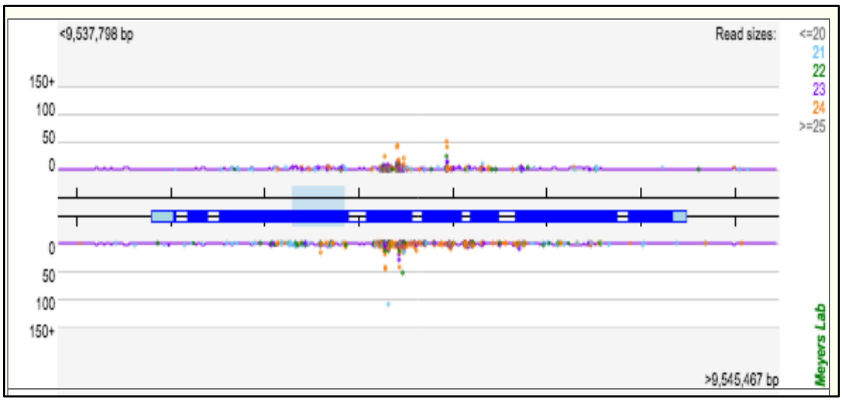

AT5G47260
(Putative Disease Resistance Protein)

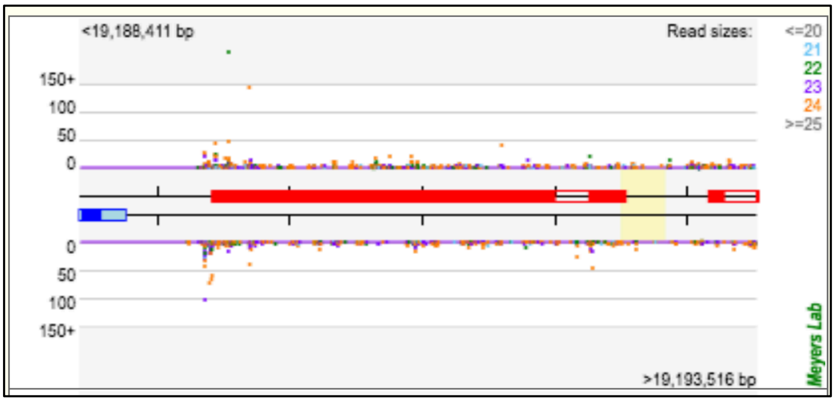

AT4G16990
(RLM3/TIR-NBS-LRR)

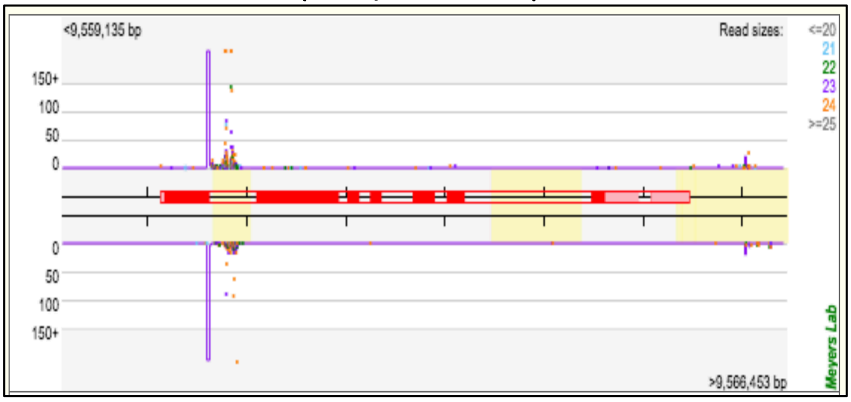

AT1G58602
(RPP7 fragment)

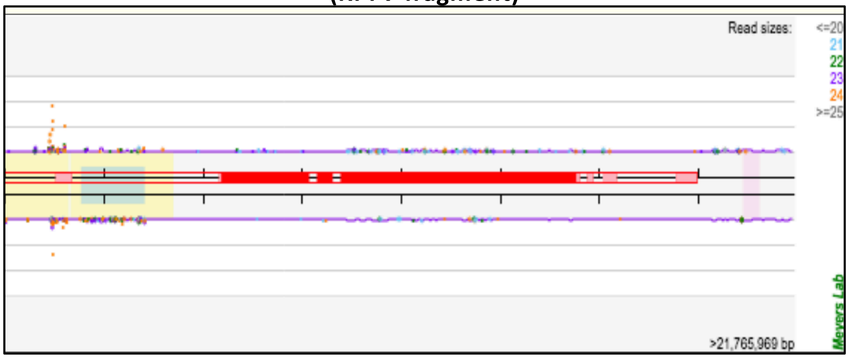

Fig. S5 SiRNA production from different NLR genes. Images show screenshots from MPSS showing sRNAs that originate from NLR genes in *Arabidopsis* selected among those displayed in Fig. 4B for accumulating the highest levels of sRNAs

Fig. S6

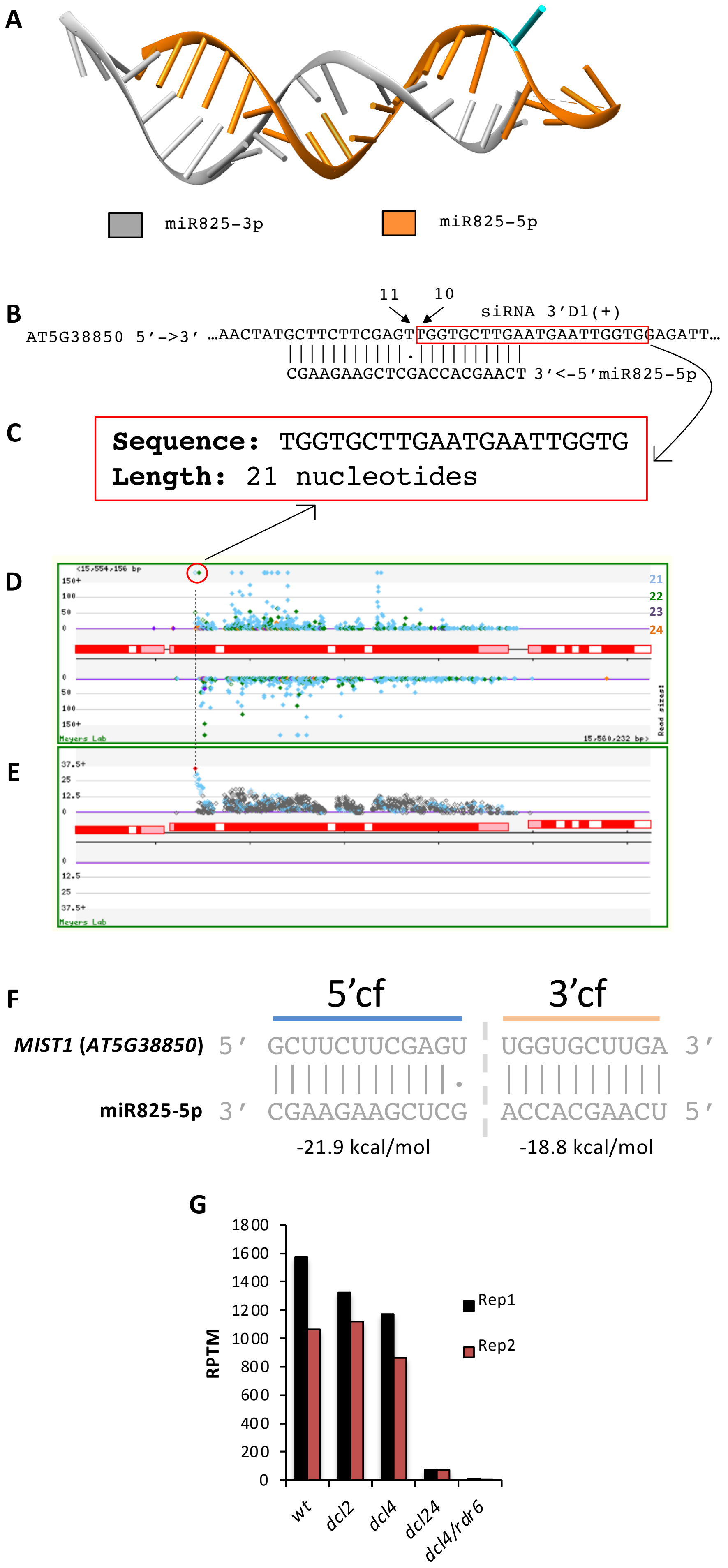
A
miR825-3p
miR825-5p
B
11
10
siRNA 3'D1(+)
AT5G38850 5'->3' …AACTATGCTTCTTCGAGTTGGTGCTTGAATGAATTGGTGGAGATT…
CGAAGAAGCTCGACCACGAACT 3'<-5'miR825-5p
C
Sequence: TGGTGCTTGAATGAATTGGTG
Length: 21 nucleotides
D
150+
100
50
0
21
22
23
24
Meyers Lab
E
37.5+
25
12.5
0
Meyers Lab
F
5'cf
3'cf
MIST1 (AT5G38850) 5' GCUUCUUCGAGU UGGUGCUUGA 3'
miR825-5p 3' CGAAGAAGCUCG ACCACGAACU 5'
-21.9 kcal/mol
-18.8 kcal/mol
G
RPTM
1800
1600
1400
1200
1000
800
600
400
200
0
Rep1
Rep2
wt
dcl2
dcl4
dcl24
dcl4/rdr6

Fig. S6 MiRNA825-5p is a trigger for phasiRNAs production from *MIST1* transcripts. A Predicted 3D-structure for the asymmetric duplex formed between miR825-5p and miR825-3p. Predictions were done using RNAfold and MC-fold/MC-Sym pipeline. **B** Sequence complementarity between miR825-5p and its target site in *MIST1*. Sequence matching the first sRNA that accumulates from this transcript is highlighted. Its position matches that predicted for the first phasiRNA (3'D1$^{(+)}$) to be generated after cleavage by RISC-miR825-5p, between nucleotides 10 and 11. **C** Sequence and length of the sRNA highlighted in **B**. **D** Screenshot from MPSS showing sRNAs that accumulate from *MIST1* (*AT5G38850)*. **E** Screenshot from MPSS showing the Phasing Analysis for the region analyzed in **D**. Each dot represents a window of ten cycles of sRNAs 21-nt in length, with the score for the degree of phasing indicated in the Y axis (scores calculated as described in (Howell *et al.*, 2007)). The red dot is the highest scoring window. Dots colored otherwise correspond to window in phase with the highest scoring (solid dots are exactly in phase, open dots are almost in phase [-1/+1]. Only 21-nt reads are considered for the analysis. **F** Predicted free energy for hybridization between miR825-5p and 3' target fragment/ 5' target fragment of *MIST1*. Prediction was done using UNAFold as previously described by (Branscheid *et al.*, 2015). **G** Quantification of sRNAs produced from *MIST1* as RPTM (Reads Per Ten Million) in two independent biological replicates in different plant mutant backgrounds.

Fig. S7

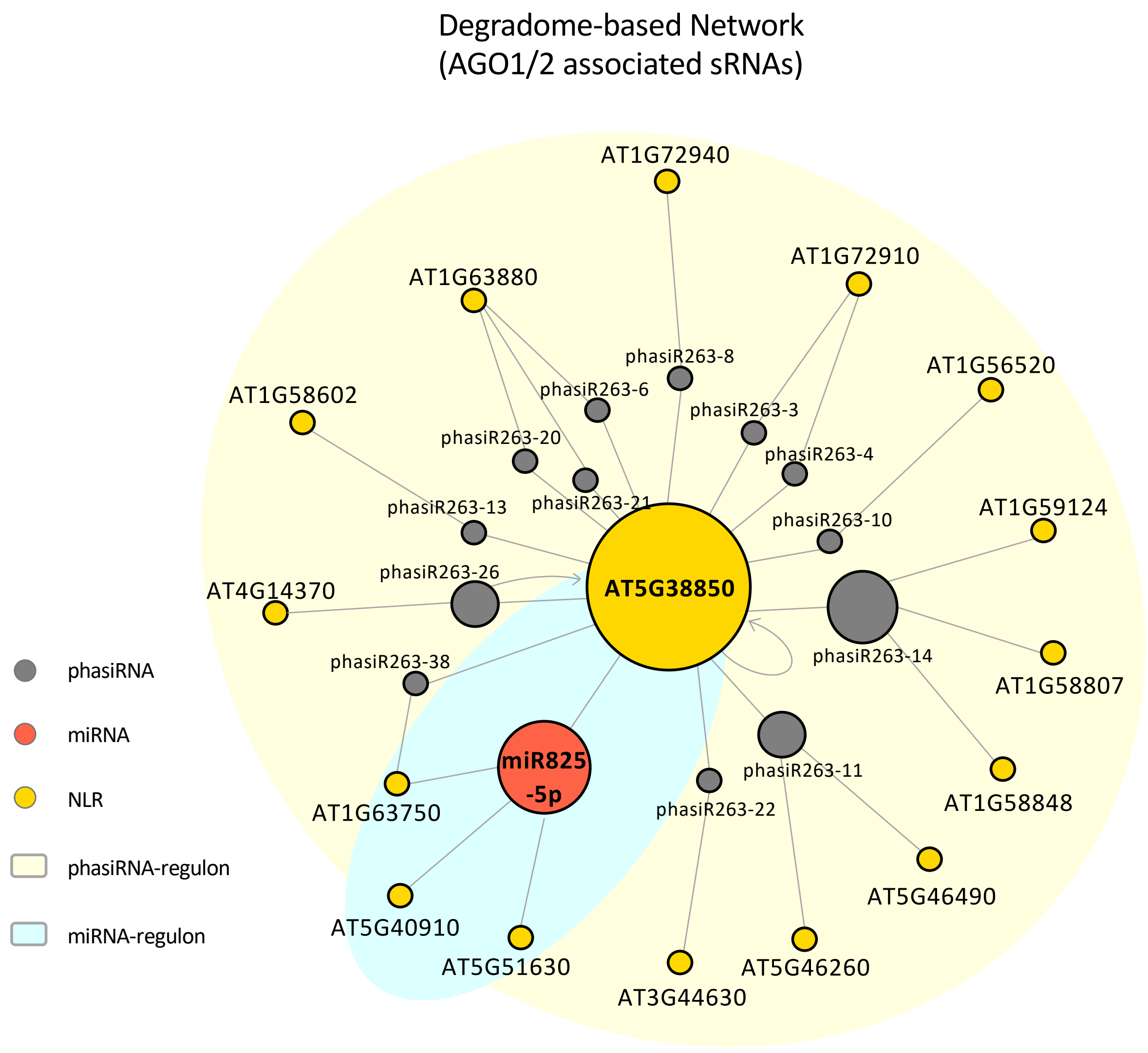

Fig. S7 Regulatory network based on degradome data for miR825-5p and *MIST1*-derived miR825-5p-triggered phasiRNAs. Network shows gene targeting based on the results detailed in Table 1, obtained following previously published reports (Zhai *et al.*, 2011; Deng *et al.*, 2018) using raw data from PARE libraries of Arabidopsis under basal conditions to look for degradome tags supporting phasiRNA targeting of TNL gene transcripts. Target genes represented include those for which tags significantly accumulated precisely map to predicted cleavage position within the phasiRNA matching sequence (Deng *et al.*, 2018) in at least two independent libraries, and after filtering out those with a peak category above 2. Size of signs indicating each phasiRNA.

Fig. S8

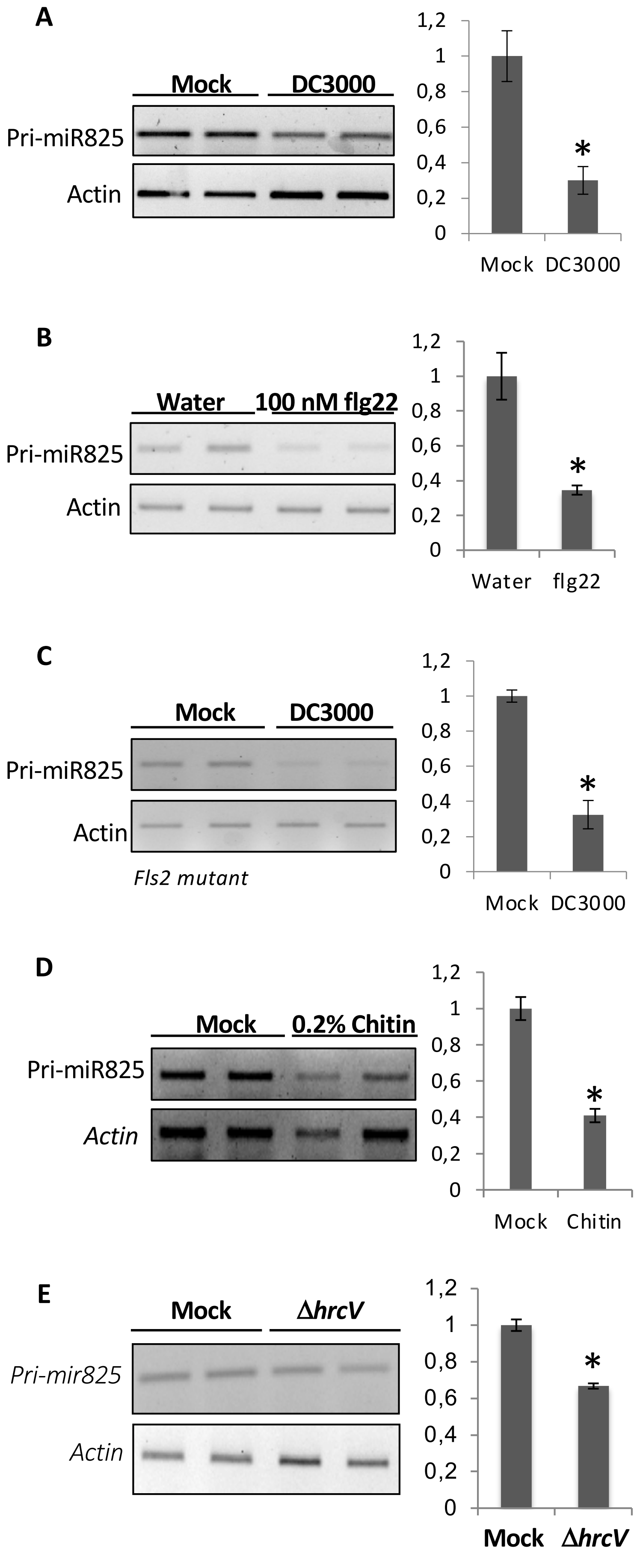

Fig. S8 Pri-miR825 is downregulated by PAMPs. A-D Semi quantitative RT-PCR show levels of pri-miR825 3 hours: **A** post-inoculation with $5x10^7$ CFU/ml of *P. syringae* DC3000. **B** post-treatment with flg22, **C** post-inoculation of *fls2* mutant plants with, $5x10^7$ CFU/ml of *P. syringae* DC3000, **D** post-treatment with chitin. Accompanying graphs correspond to Image J quantification of the bands relative to actin, and **E** post-inoculation with $5x10^7$ CFU/ml of *P. syringae* DC3000.

Fig. S9

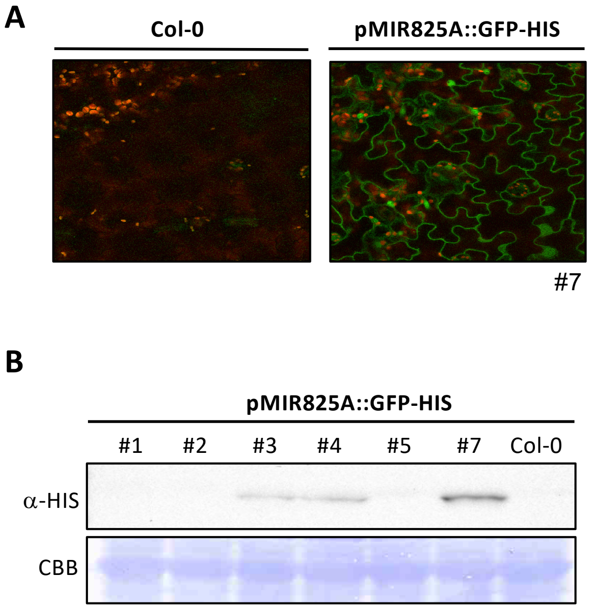

Fig. S9 *A*tmiR825A promoter is active in adult leaves. A Confocal microscopy images showing GFP accumulation in a transgenic line harbouring the reporter gene under the control of *At*miR825 promoter (pMIR825A::GFP-HIS). **B** Western blot analysis using anti-HIS antibody show accumulation of the GFP-HIS reporter fusion protein in several transgenic lines harbouring the pMIR825A::GFP-HIS construct. The membrane was stained with Coomassie and used as loading control. Samples for **a** and **b** were taken from *Arabidopsis* adult leaves.

Fig. S10

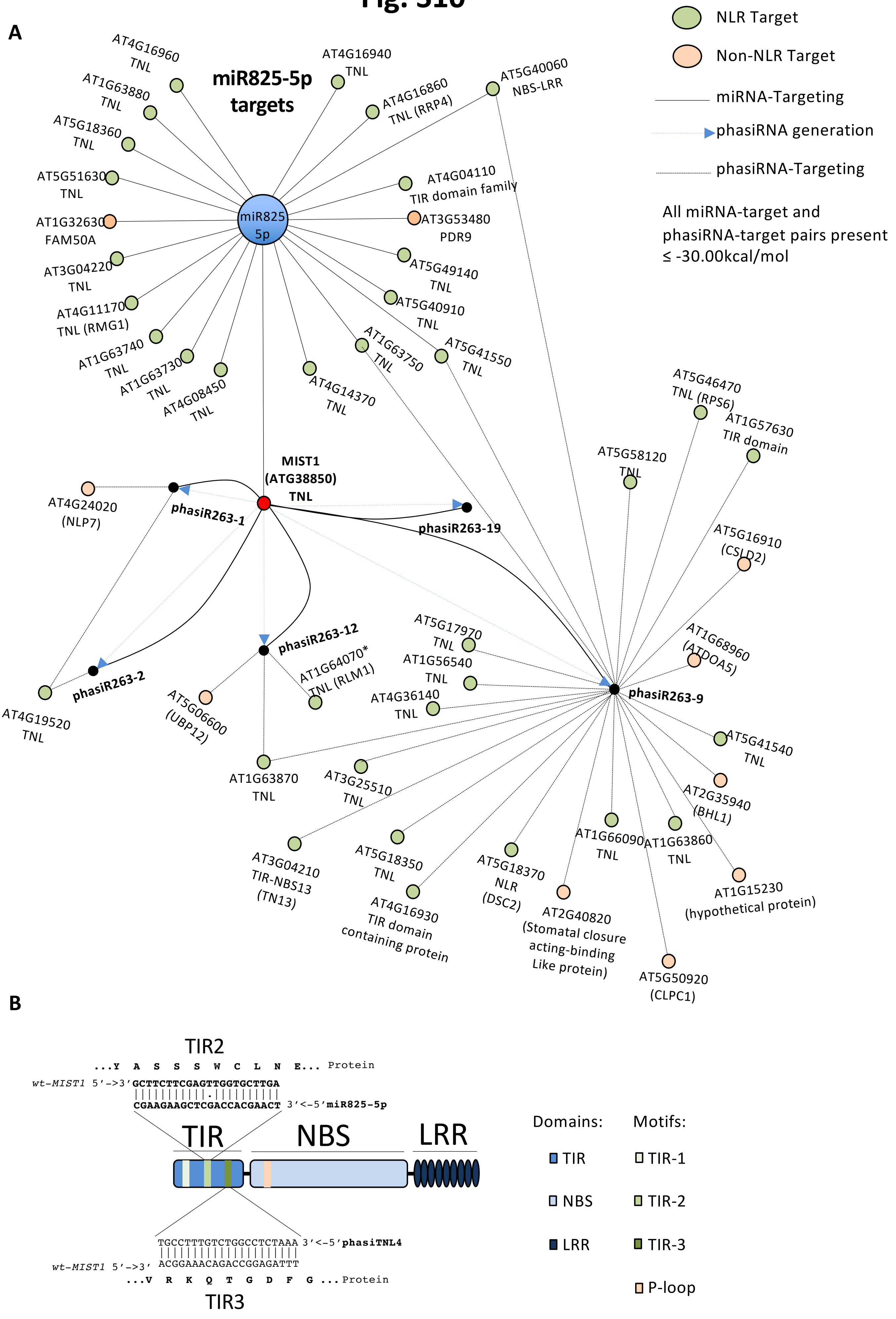
A
miR825-5p targets
miR825 5p
NLR Target
Non-NLR Target
miRNA-Targeting
phasiRNA generation
phasiRNA-Targeting
All miRNA-target and phasiRNA-target pairs present ≤ -30.00kcal/mol
AT4G16960 TNL
AT1G63880 TNL
AT5G18360 TNL
AT5G51630 TNL
AT1G32630 FAM50A
AT3G04220 TNL
AT4G11170 TNL (RMG1)
AT1G63740 TNL
AT1G63730 TNL
AT4G08450 TNL
AT4G14370 TNL
AT1G63750 TNL
AT5G41550 TNL
AT5G40910 TNL
AT5G49140 TNL
AT3G53480 PDR9
AT4G04110 TIR domain family
AT4G16860 TNL (RRP4)
AT4G16940 TNL
AT5G40060 NBS-LRR
MIST1 (ATG38850) TNL
AT4G24020 (NLP7)
phasiR263-1
phasiR263-19
phasiR263-2
phasiR263-12
phasiR263-9
AT4G19520 TNL
AT5G06600 (UBP12)
AT1G64070* TNL (RLM1)
AT1G63870 TNL
AT5G17970 TNL
AT1G56540 TNL
AT4G36140 TNL
AT3G25510 TNL
AT3G04210 TIR-NBS13 (TN13)
AT5G18350 TNL
AT4G16930 TIR domain containing protein
AT5G18370 NLR (DSC2)
AT2G40820 (Stomatal closure acting-binding Like protein)
AT1G66090 TNL
AT1G63860 TNL
AT5G50920 (CLPC1)
AT1G15230 (hypothetical protein)
AT2G35940 (BHL1)
AT5G41540 TNL
AT1G68960 (ATDOA5)
AT5G16910 (CSLD2)
AT5G58120 TNL
AT1G57630 TIR domain
AT5G46470 TNL (RPS6)
B
TIR2
...Y A S S S W C L N E... Protein
wt-MIST1 5'->3' GCTTCTTCGAGTTGGTGCTTGA
CGAAGAAGCTCGACCACGAACT 3'<-5' miR825-5p
TIR
NBS
LRR
TGCCTTTGTCTGGCCTCTAAA 3'<-5' phasiTNL4
wt-MIST1 5'->3' ACGGAAACAGACCGGAGATTT
...V R K Q T G D F G ...Protein
TIR3
Domains:
TIR
NBS
LRR
Motifs:
TIR-1
TIR-2
TIR-3
P-loop

Fig. S10 MiR825-5p predicted regulatory network. A MiR825-5p direct target genes are shown including MIST1 as a primary central hub for TNL gene regulation. Only the top five phasiTNLs in terms of accumulation and AGO1/AGO2 association are included, as well as their predicted targets. Hits of this phasiTNLs on primary miR825-5p are also indicated. PhasiTNL4 acts as a secondary hub in this regulatory network through targeting of the highly conserved TIR3 motif. **B** Domain organization of a TNL protein with domains and conserved motifs indicated. Alignments of miR825-5p and phasiTNLs with their respective target sequences are mapped to the corresponding domains/motifs within the TNL protein.

Table S1. Primers used in this study

Name	Sequence	Used to
amiR825-5p-I	GATCAAGCACCAGCTCGAAGAAGCTCTCTCTTTTGTATTCC	Increase levels of miR825-5p
amiR825-5p-II	GAGCTTCTTCGAGCTGGTGCTTGATCAAAGAGAATCAATGA	Increase levels of miR825-5p
amiR825-5p-III	GAGCCTCTTCGAGCTGTGCTTGATCACAGGTCGTGATATG	Increase levels of miR825-5p
amiR825-5p-IV	GATCAAGCACAGCTCGAAGAGGCTCTACATATATATTCCT	Increase levels of miR825-5p
amiR-A	CTGCAAGGCGATTAAGTTGGGTAAC	For cloning amiRs
amiR-B	GCGGATAACAATTTCACACAGGAAACAG	For cloning amiRs
STTM825-5p-F	AAGTTAACGCTTCTTCGAGCCTATGGTGCTTGAGTTGTTGTTGTTATGGTCTAATTTAAATATGGTCT	Reduce levels of miR825-5p
STTM825-5p-R	TTGGATCCTCAAGCACCATAGGCTCGAAGAAGCATTCTTCTTCTTTAGACCATATTTAAATTAGACCA	Reduce levels of miR825-5p
amiR-anti825-I	GATAAGCTTCTTCGAGCTGGTGCTTCTCTCTTTTGTATTCC	Reduce levels of Pri-miR825
amiR-anti825-II	GAAGCACCAGCTCGAAGAAGCTTATCAAAGAGAATCAATGA	Reduce levels of Pri-miR825
amiR-anti825-III	GAAGAACCAGCTCGATGAAGCTTATCACAGGTCGTGATATG	Reduce levels of Pri-miR825
amiR-anti825-IV	GATAAGCTTCATCGAGCTGGTTCTTCTACATATATATTCCT	Reduce levels of Pri-miR825
pro825-2kb-F	TAAAGCGGCCGCATGCTGTTTTCCAATAAAGTTAATTC	For cloning the promoter of AthmicroRNA825A
pro825-2kb-R	AAGGCGCGCCTATTTTCTAAGATAAATTAGCTAAGC	For cloning the promoter of AthmicroRNA825A
Pri-miR825-qPCR-F	ACTCGTTCAAGCACCAGCTC	Cuantification of Pri-miR825
Pri-miR825-qPCR-R	CATCAACTTGTTCATGCACCTT	Cuantification of Pri-miR825
MIST1-qPCR-F	GAGAGGAGCCAAACCATAGC	Cuantification of MIST1 (TIR-NBS-LRR)
MIST1-qPCR-R	TCGGCATCACTACGTCTTTGC	Cuantification of MIST1 (TIR-NBS-LRR)
Actin2-qPCR-F	ACTAAAACGCAAAACGAAAGCGGTT	Cuantification of Actin2/q-PCR normalization
Actin2-qPCR-F	CTAAGCTCTCAAGATCAAAGGCTTA	Cuantification of Actin2/q-PCR normalization
Reverse-universal-stemloop q-	GTGCAGGGTCCGAGGT	Cuantification of miRNAs
RT stem-loop miR825-5p	GTCGTATCCAGTGCAGGGTCCGAGGTATTCGCACTGGATACGACGCTTCT	For pulsed RT and cuantification of miR825-5p
miR825-5p stem-loop qPCR F	TGGCTCAAGCACCAGCTCGA	Cuantifiation of miR825-5p
U6 probe	GCTAATCTTCTCTGTATCGTTCC	Northern blot loading control
miR825-5p probe	GCTTCTTCGAGCTGGTGCTTGA	miR825-5p detection by Northern blot
MIST1_PhasiRNA_probeF	TTTGAAATGCAAAGACGTAG	Generation of long probe for phasiRNA detection by northern blot
MIST1_PhasiRNA_probeR	TTGGCGCGCCCTTTCATATTCATTTCTCCTTTCACTG	Generation of long probe for phasiRNA detection by northern blot
MIGS825-5p-TS F	AAGCGGCCGCGCTTCTTCGAGTTGGTGCTTGATCTTCTCTAGCCGTGGTCGTC	Generation of MIGS
MIGS825-5p-TS R	AAGGCGCGCCGGCCATTTCCTTCAGCCTATAT	Generation of MIGS

Supplementary Table 2. ACT2 expression relative to UBQ5 in miR825-5p altered genotypes and statistical analysis.

Genotype	Actin	Average	SD	SE
Col-0	1<br>1.43064593<br>1.08172467	1.1707902	0.22872141	0.132052366
STTM3	0.76666417<br>1.74512858<br>0.58777395	1.0331889	0.62301204	0.35969617
STTM4	0.88679139<br>0.96593633<br>0.86653705	0.90642159	0.05252678	0.030326352
OX3	1.104454<br>1.94980971<br>2.12382761	1.72603044	0.54528755	0.314821912
OX12	0.80478017<br>0.79186881<br>0.70222244	0.76629047	0.05585885	0.032250119

ANOVA summary	
F	2.764
P value	0.0876
P value summary	ns
Significant diff. among means (P < 0.05)?	**NO**
R square	0.5251

Tukey's multiple comparisons test	Mean Diff.	95.00% CI of diff.	Significant?	Summary	Adjusted P Value
Col-0 vs. STTM3	0.1376	-0.8987 to 1.174	**NO**	ns	0.9912
Col-0 vs. STTM4	0.2644	-0.7720 to 1.301	**NO**	ns	0.9121
Col-0 vs. OX3	-0.5552	-1.592 to 0.4811	**NO**	ns	0.4423
Col-0 vs. OX12	0.4045	-0.6318 to 1.441	**NO**	ns	0.7058
STTM3 vs. STTM4	0.1268	-0.9096 to 1.163	**NO**	ns	0.9936
STTM3 vs. OX3	-0.6928	-1.729 to 0.3435	**NO**	ns	0.2544
STTM3 vs. OX12	0.2669	-0.7694 to 1.303	**NO**	ns	0.9093
STTM4 vs. OX3	-0.8196	-1.856 to 0.2167	**NO**	ns	0.1431
STTM4 vs. OX12	0.1401	-0.8962 to 1.176	**NO**	ns	0.9906
OX3 vs. OX12	0.9597	-0.07659 to 1.996	**NO**	ns	0.0729

Supplementary Table 3. ACT2 expression relative to UBQ5 after flg22 treatment and statistical analysis.

Treatment	Actin	Average	SD	SE	*t*-test P value	P value summary	Significantly different (P < 0.05)?
	0.41561895						
Mock 0 h	1	0.65895	0.304199	0.17563			
	0.56123102				0.1942	ns	**NO**
	0.82074161						
Flg22 0 h	0.97490486	0.9549822	0.125471	0.07244			
	1.0693						
	1						
Mock 3 h	1.21139274	1.0822192	0.113249	0.06538			
	1.03526492				0.3916	ns	**NO**
	0.44084192						
Flg22 3 h	0.9794203	0.8634635	0.378218	0.21836			
	1.17012825						
	1						
Mock 6 h	1.50350882	1.1744474	0.285148	0.16463			
	1.01983329				0.2832	ns	**NO**
	1.17419064						
Flg22 6 h	0.62057035	0.8901182	0.277096	0.15998			
	0.87559361						
	1						
Mock 14 h	2.3456699	1.7119067	0.67623	0.39042			
	1.79005014				0.0636	ns	**NO**
	0.61985385						
Flg22 14h	0.75262337	0.7112819	0.0793	0.04578			
	0.76136844						

Table S4. Antibodies used in this work

Antibody	Working dilution	Reference
anti-GFP	1:600	Santa Cruz Biotechnology, USA
anti-Tubulin	1:1000	Abiocode M0267
anti-MPKs	1:5000	Cell Signaling Biotech #4370
anti-PR1	1:5000	(Wang *et al.*, 2005)*
anti-Rabbit	1:10000	SIGMA A6154
anti-Mouse	1:80000	SIGMA A9044

*Wang D, Weaver ND, Kesarwani M, Dong X. 2005. Induction of protein secretory pathway is required for systemic acquired resistance. *Science* 308(5724): 1036-1040.

Table S5. Bacterial strains used in this work

Strain	Reference	Antibiotic selection*
Pseudomonas syringae pv. tomato DC3000	Cuppels, 1986[1]	
Pseudomonas syringae pv. tomato DC3000 constitutively expressing AvrPt2	Macho *et al.* 2009[2]	15 µg/ml kanamycin (pAME)
Agrobacterium tumefaciens C58C1	Deblaere *et al.* 1985	50 µg/ml rifampicin (genomic)<br>5 µg/ml tetracycline (helper plasmid PGV2260)<br>50 µg/ml kanamycin (pGWB2, pBINX)<br>40 µg/ml hygromycin (pGWB2, pMDC111)
Agrobacterium tumefaciens GV3101	Koncz *et al.* 1994	50 µg/ml rifampicin (genomic)<br>5 µg/ml tetracycline (helper plasmid pSOUP)<br>50 µg/ml kanamycin (pGWB2, pBINX)<br>40 µg/ml hygromycin (pGWB2, pMDC111)<br>20 µg/ml gentamycin (helper plasmid pMP90)

*Genomic resistance or plasmid resistance when indicated

[1]Cuppels, D. A. 1986. Generation and Characterization of Tn*5* Insertion Mutations in *Pseudomonas syringae* pv. tomato. Appl. Environ. Microbiol. 51:323-327.

[2]Macho AP, Ruiz-Albert J, Tornero P, Beuzón CR. 2009 Identification of new type III effectors and analysis of the plant response by competitive index. Mol Plant Pathol. 10:69–80.

[3]Deblaere R, Bytebier B, De Greve H, Deboeck F, Schell J, Van Montagu M, et al. 1985 Efficient octopine Ti plasmid-derived vectors for *Agrobacterium*-mediated gene transfer to plants. Nucleic Acids Res.13:4777–88.

[4]Koncz C., Martini N., Szabados L., Hrouda M., Bachmair A., Schell J. (1994) Specialized vectors for gene tagging and expression studies. In: Gelvin S.B., Schilperoort R.A. (eds) Plant Molecular Biology Manual. Springer, Dordrecht.

Table S6. Plasmids generated/used in this study

Plasmid	Cloned	Used to	Promoter	Plant Resistance
pGEM-T	amiR825-5p	Cloning of amiR825-5p	-	-
pBINX`	amiR825-5p	Increases levels of miRNA825-5p (22nt)	35S	Km
pBINX`	amiR319	Increases levels of miR319	35S	Km
pBINX`	STTM825-5p	Reduce levels of miRNA825-5p (22nt)	35S	Km
pBINX`	Empty Vector	Control	35S	Km
pBSKII	anti-825	Cloning of anti-825	-	-
pBINX`	anti-825	Reduce levels of Pri-miR825	35S	Km
pENTR	microRNA825A 2kb promoter	Cloning of microRNA825A 2kb promoter	-	-
pMDC111	microRNA825A 2kb promoter	Reporter of micoRNA825A promoter activity with GFP	-	Hyg
pENTR	*AT5GG38850* genomic region	Cloning of *AT5GG38850* genomic region	-	-
PEG103	*AT5GG38850* genomic region	Increases levels of *AT5G38850* fused to GFP	35S	Basta
pENTR	*AT5GG38850* genomic region with a mutated miR825-5p target site	Cloning of *AT5GG38850* genomic region with a mutated miR825-5p target site	-	-
PEG103	*AT5GG38850* genomic region with a mutated miR825-5p target site	Increases levels of a miR825-5p resistant version of *AT5G38850* fused to GFP	35S	Basta
pENTR	MIGS825-5p-TS	Cloning of MIGS825-5p-TS	-	-
pGWB2	MIGS825-5p-TS	Produce a fusion between miR825-5p Target site and a fragment of *AGAMOUS* gene	35S	Km and Hyg

Table S7. Transgenic lines used in this study

Transgenic lines (*A. thaliana*)	Plasmid used	Plant resistance/selection	Description
amiR anti-825	pBINX'	Km	Transgenic line expressing an artificial miRNA (amiR) against Pri-miR825
amiR825-5p	pBINX'	Km	Transgenic line that increase miR825-5p levels
STTM825-5p	pBINX'	Km	Transgenic line that reduce miR825-5p levels
MIGS825-5p-TS	pGWB2	Km/Hyg	Transgenic line harbouring a fusion between miR825-5p Target site and a fragment of *AGAMOUS* gene
MIGS825-5p-TS x amiR825-5p	pGWB2/pBINX'	Km/Hyg	Cross between both transgenic line described above
Pro825::GFP	pMDC111	Hyg	Transgenic line harbouring a phusion between miR825 promoter and GFP reporter

Table S8. Software used in this work

Software	Purpose	Data source	Parameters	Reference
SRA toolkit fastq-dump	Raw file retrival and conversion to fasq files	Sequence Read Archive (NCBI)	Standard/default parameters	http://ncbi.github.io/sra-tools/
Trimmomatic	Quality filtered applied for adapter removal	Fastq files obtained using fastq-dump	Standard/default parameters	(Bolger *et al.*, 2014)[1]
FASTQC	Quality confirmation	Fastq files quality-filetered by Trimmomatic	Standard/default parameters	http://www.bioinformatics.babraham.ac.uk/projects/fastqc/
Bowtie	Mapping of reads to Arabidopsis genome (TAIR10). SAM output.	Reads from FASTQC-confirmed fastq files	No mismatches (–v 0 mode), except for AGO1 and AGO2 libraries (-v 1 mode)	(Langmead *et al.*, 2009)[2]

samtools	Convertion to BAM, sorting and indexing	SAM files	Standard/default parameters	(Li *et al.*, 2009)[3]
IGV browser	Reads visualization	Sorted and indexed BAM files	Standard/default parameters	(Robinson *et al.*, 2011)[4]
HTSeq htseq-count	Estimating numbers of reads mapped per feature of the *A. thaliana* genome	SAM and GFF files	Normalization to ten millions of total reads mapped to the entire genome (RPTM method)	(Anders *et al.*, 2015) [5]
strucVis	Display small RNA coverage and secondary structure of ath-miR825 precursor	Raw reads retrieved from SRR2079800	Standard/default parameters	https://github.com/MikeAxtell/strucVis
WMD3	microRNA and siRNA target prediction	miRNA / siRNA fasta files	Standard/default parameters	(http://wmd3.weigelworld.org/cgi-bin/webapp.cgi; Ossowski Stephan, Fitz Joffrey, Schwab Rebecca, Riester Markus and Weigel Detlef, personal communication)

psRNATarget	microRNA and siRNA target prediction	miRNA / siRNA fasta files	Standard/default parameters	(Dai & Zhao, 2011)[6]
Clustal Omega	To analyze miRNA825 conservation	Sequences retrieved from miRBase or NCBI (Blastn against the genomes with ath-miR825 as template)	Standard/default parameters	(Madeira *et al.*, 2019)[7]<br>miRBase (Griffiths-Jones *et al.*, 2006)[8]
Weblogo	Generation of logos	NLR sequences were retrieved from TAIR	Standard/default parameters	(Crooks *et al.*, 2004) [9]
"grep" UNIX / Linux command	Determination of 5p and 3p read number for	Raw files obtained from	Only reads starting with mature miRNA sequences and containing the adapter	N/A

	calculation of miR825 5p/3p ratios.	Sequence Read Archive (NCBI) under the accession numbers listed in **Table S6,** converted to fastq files	sequence immediately after miRNA reads were used. Ratios were calculated for each library	
CleaveLand4	Degradome analyses against *Arabidopsis thaliana* cDNA NLR-transcriptome	Four degradome libraries (**Table S6**) analyzed against AGO-bound sRNAs. Just sRNAs with ≥5 of mapped reads	Bowtie parameters for modified to -v 0 [no mismatch allowed] and -k 10 [report up to 10 valid alignments] during alignment. Output was filtered retaining only degradome categories 0-2 and Allen scores 0-6.<br><br>Only sRNA-NLR interactions detected in at least 2 of the	(Addo-Quaye *et al.*, 2009)[10]<br>Output filtering (Gyula *et al.*, 2018)[11]<br>Ws/Wl ratios (Zhai *et al.*, 2011)[12]

		were used in the analysis.	libraries were considered significative.<br><br>Ws/Wl ratios: number of PARE reads within a small window (5 nt ±2) divided by the number of reads within a large window (31 nt ±15), considered as validated when equal or larger than 0.5	
Mfold	Prediction of secondary RNA structure and Vienna format.	fastq file	Standard/default parameters	(Zuker, 2003)[13]
Varna	Visualization of secondary RNA structure	Vienna format	Standard/default parameters	(Darty *et al.*, 2009)[14]
Mfold, RNAhybrid	Prediction of secondary and tertiary structures	Fasta	Standard/default parameters	Mfold (Zuker, 2003)[13]<br>RNAhybrid (Krüger *et al.*, 2006)[15]<br>MC-fold/MC-sym (Parisien & Major, 2008)[16]

and MC-fold/MC-Sym	for the miR8255-p/miR825-3p duplex			Overall pipeline (Manavella *et al.*, 2012)[17]

[1]Bolger AM, Lohse M, Usadel B. 2014 Trimmomatic: a flexible trimmer for Illumina sequence data. Bioinformatics 30:2114–20.
[2]Langmead B, Trapnell C, Pop M, Salzberg SL. Ultrafast and memory-efficient alignment of short DNA sequences to the human genome. Genome Biol. 2009; 10:R25–10.
[3]Li H, Handsaker B, Wysoker A, Fennell T, Ruan J, Homer N, et al. 2009 The Sequence Alignment/Map format and SAMtools. Bioinformatics. 25:2078–9.
[4]Robinson JT, Thorvaldsdóttir H, Winckler W, Guttman M, Lander ES, Getz G, et al. 2011 Integrative genomics viewer. Nat Biotechnol. 29:24–6.
[5]Anders S, Pyl PT, Huber W. 2015 HTSeq--a Python framework to work with high-throughput sequencing data. Bioinformatics. 31:166–9.
[6]Dai X, Zhao PX. 2011 psRNATarget: a plant small RNA target analysis server. Nucleic Acids Res. 39:W155–9.
[7]Madeira F, Park YM, Lee J, Buso N, Gur T, Madhusoodanan N, et al. 2019 The EMBL-EBI search and sequence analysis tools APIs in 2019. Nucleic Acids Res. 47:W636–41.
[8]Griffiths-Jones S, Grocock RJ, van Dongen S, Bateman A, Enright AJ. 2006 miRBase: microRNA sequences, targets and gene nomenclature. Nucleic Acids Res. 34:D140–4.
[9]Crooks GE, Hon G, Chandonia J-M, Brenner SE. 2004 WebLogo: a sequence logo generator. Genome Res; 14:1188–90.
[10]Addo-Quaye C, Miller W, Axtell MJ. 2009. CleaveLand: a pipeline for using degradome data to find cleaved small RNA targets. Bioinformatics 25: 130–131.
[11]Gyula P, Baksa I, Tóth T, Mohorianu I, Dalmay T, Szittya G. 2018. Ambient temperature regulates the expression of a small set of sRNAs influencing plant development through NF- YA2and YUC2. Plant, Cell & Environment 41: 2404–2417.
[12]Zhai J, Jeong DH, De Paoli E, Park S, Rosen BD, Li Y, Gonzalez AJ, Yan Z, Kitto SL, Grusak MA, Jackson SA, Stacy G, Cook DR, Green PJ, Sherrier DJ, Meyers BC. 2011. MicroRNAs as master regulators of the plant NB-LRR defense gene family via the production of phased, trans-acting siRNAs. Genes & Development 25: 2540–2553.
[13]Zuker M. 2003. Mfold web server for nucleic acid folding and hybridization prediction. Nucleic Acids Research 31: 3406–3415.
[14]Darty K, Denise A, Ponty Y. 2009. VARNA: Interactive drawing and editing of the RNA secondary structure. Bioinformatics 25: 1974–1975.
[15]Kruger J, & Rehmsmeier M (2006). RNAhybrid: microRNA target prediction easy, fast and flexible. Nucleic Acids Res, 34, W451-4.
[16]Parisien M, Major F. The MC-Fold and MC-Sym pipeline infers RNA structure from sequence data. Nature. 2008 Mar 6;452(7183):51-5. doi: 10.1038/nature06684. PMID: 18322526.

[17]Manavella PA, Koenig D, Weigel D. 2012. Plant secondary siRNA production determined by microRNA-duplex structure. Proceedings of the National Academy of Sciences of the United States of America 109: 2461–2466.

Table S9: Libraries used in this work

Accession number	Description
GSM2787769<br>GSM2787770<br>GSM3909547<br>GSM3909548	**AGO1/AGO2-loaded small RNA IP libraries**
SRR1171802<br>SRR1171803<br>SRR1171804<br>SRR10322040	**Degradome libraries**<br>[dataset]. Arabidopsis thaliana, omics_ena_project, V1; 1970. https://www.ncbi.nlm.nih.gov/bioproject/?term=PRJNA238653.
SRR2079799<br>SRR2079800<br>SRR2079801<br>SRR2079802<br>SRR2079803<br>SRR2079804<br>SRR2079805<br>SRR2079806<br>SRR2079807<br>SRR2079808<br>SRR2079809<br>SRR2079810<br>SRR2079811<br>SRR2079812	**Sequence Read Archive (NCBI) files used to determine miR825 5p/3p ratios**<br>Submitted by: Gene Expression Omnibus (GEO)<br>Study: Time-course transcriptome of wild-type Arabidopsis leaf
